# Genome analysis of *E. coli* isolated from Crohn’s disease patients

**DOI:** 10.1186/s12864-017-3917-x

**Published:** 2017-07-19

**Authors:** Daria V. Rakitina, Alexander I. Manolov, Alexandra V. Kanygina, Sofya K. Garushyants, Julia P. Baikova, Dmitry G. Alexeev, Valentina G. Ladygina, Elena S. Kostryukova, Andrei K. Larin, Tatiana A. Semashko, Irina Y. Karpova, Vladislav V. Babenko, Ruzilya K. Ismagilova, Sergei Y. Malanin, Mikhail S. Gelfand, Elena N. Ilina, Roman B. Gorodnichev, Eugenia S. Lisitsyna, Gennady I. Aleshkin, Petr L. Scherbakov, Igor L. Khalif, Marina V. Shapina, Igor V. Maev, Dmitry N. Andreev, Vadim M. Govorun

**Affiliations:** 1 Federal Research and Clinical Center of Physical-Chemical Medicine of Federal Medical Biological Agency, Moscow, Russia; 2 Moscow Institute for Physics and Technology, Moscow, Russia; 3 Skolkovo Institute of Science and Technology, Moscow, Russia; 4 A.A. Kharkevich Institute for Information Transmission Problems, Russian Academy of Sciences, Moscow, Russia; 5 Institute of Fundamental Medicine and Biology of Kazan Federal University, Kazan, Russia; 6 Faculty of Computer Science, National Research University Higher School of Economics, Moscow, Russia; 7 Faculty of Bioengineering and Bioinformatics, Moscow State University, Moscow, Russia; 8 The Gamaleya Research Institute for Epidemiology and Microbiology of the Russian Academy of Medical Science, Moscow, Russia; 9 Central Scientific Institute of Gastroenterology, Moscow Clinical Research Centre, Moscow, Russia; 10 State Scientific Center of Coloproctology, Ministry of Health of Russian Federation, Moscow, Russia; 11 Moscow State University of Medicine and Dentistry named after A.I. Evdokimov, Ministry of Health of Russian Federation, Moscow, Russia

**Keywords:** Crohn’s disease, *E. coli*, Genome, Propanediol

## Abstract

**Background:**

*Escherichia coli* (*E. coli*) has been increasingly implicated in the pathogenesis of Crohn’s disease (CD). The phylogeny of *E. coli* isolated from Crohn’s disease patients (CDEC) was controversial, and while genotyping results suggested heterogeneity, the sequenced strains of *E. coli* from CD patients were closely related.

**Results:**

We performed the shotgun genome sequencing of 28 *E. coli* isolates from ten CD patients and compared genomes from these isolates with already published genomes of CD strains and other pathogenic and non-pathogenic strains. CDEC was shown to belong to A, B1, B2 and D phylogenetic groups. The plasmid and several operons from the reference CD-associated *E. coli* strain LF82 were demonstrated to be more often present in CDEC genomes belonging to different phylogenetic groups than in genomes of commensal strains. The operons include carbon-source induced invasion GimA island, prophage I, iron uptake operons I and II, capsular assembly pathogenetic island IV and propanediol and galactitol utilization operons.

**Conclusions:**

Our findings suggest that CDEC are phylogenetically diverse. However, some strains isolated from independent sources possess highly similar chromosome or plasmids. Though no CD-specific genes or functional domains were present in all CD-associated strains, some genes and operons are more often found in the genomes of CDEC than in commensal *E. coli*. They are principally linked to gut colonization and utilization of propanediol and other sugar alcohols.

**Electronic supplementary material:**

The online version of this article (doi:10.1186/s12864-017-3917-x) contains supplementary material, which is available to authorized users.

## Background

Crohn’s disease (CD), one of the major forms of inflammatory bowel disease (IBD), is a chronic generalized inflammation of the gastrointestinal tract. The histological picture of Crohn’s disease includes thickened submucosa, transmural inflammation, fissuring ulceration, and non-caseating granulomas. The common complications in the intestine are presented by strictures, abscesses, fistulas and, in the long run, colon cancer. Extraintestinal complications include arthritis, erythema nodosum, uveitis, and primary sclerosing cholangitis.

Many factors, both genetic and environmental, are regarded to contribute to the CD pathogenesis. It is a general notion that CD is a result of abnormal immune response of genetically susceptible individuals to the imbalance in the intestinal microbiota (reviewed in [1, 2]). Host susceptibility factors include intestinal barrier dysfunctions (decreased levels of antimicrobial peptides defensins, discontinuous tight junctions and aberrant mucin assembly) and defects in innate immunity, autophagy, and phagocytosis. Polymorphisms in certain genes, e. g. *NOD2*, *ATG16L1*, and *IRGM*) involved in these processes have been reported to be associated with CD (reviewed in [1]). There are at least 71 susceptibility loci identified by genome-wide association studies, that are considered to be involved in the pathogenesis of Crohn’s disease [3].

Among dysbioses in CD patients 10–100 fold increase in abundance of *Escherichia coli* is often observed as compared to healthy individuals [4–8], so this led to several studies of *E. coli* isolated from those patients. The obtained strains were defined as pathotype adherent-invasive *E. coli* (AIEC) due to their ability to adhere and invade epithelial cells of the intestine [9, 10]. They are also able to survive and replicate within macrophages, and are selectively favored by impaired autophagy to replicate intracellularly [11]. In comparison with commensal *E. coli*, AIEC are more often resistant to antibiotics [12], and are strong biofilm producers [13]. Some of the isolated strains were shown to induce chronic inflammation by colonizing mice intestine [14, 15]. An adhesion-invasion model was proposed, according to which interaction between bacterial porin OmpC (outer membrane protein C) and human CEACAM6 (carcinoembryonic antigen related cell adhesion molecule 6) receptor were a key step in the pathogenesis [16].

Published observations on the phylogenetic diversity of the AIEC group are controversial. In some independent studies performed using various techniques including genomic hybridization assays, RAPD-PCR and serotyping, and phylotyping by a multiplex PCR protocol, *E. coli* strains from different patients were shown to be highly heterogeneous and were assigned to several phylogroups (A,B1,B2,D) [5, 13, 16–24]. The ribotyping analysis on the contrary leads to the suggestion that the majority of CDEC have evolved from the same ancestral strain from phylogroup B2 [4], perhaps by acquisition of additional virulence factors via mobile elements transfer or insertion of a pathogenicity island(s) into the bacterial chromosome [25, 26].

Results of whole-genome shotgun sequencing supported the single ancestor hypothesis. To date, four complete genomes of *E. coli* isolated from CD patients have been sequenced. They all belong to phylogroup B2. Although, these isolates were obtained from independent clinics (NRG857c from Canada [27], LF82 from Germany [28], UM146 from France [29], and HM605 [30] from United Kingdom), their genomes showed considerable sequence similarity and synteny (more than 99% sequence identity at 93–99% genome coverage). Several pathogenic islands were observed in these genomes [27, 28] as well as plasmids homologous to those from *Klebsiella* and *Salmonella* [28, 29]. However, no comparative analysis of these plasmids was performed.

In a recent paper B2-phylogroup *E. coli* genomes from CD patients were compared with 25 strains from patients with ulcerative colitis (UC) and non-IBD, and the phylogenetic heterogeneity of AIEC and CD strains was established [31]. No gene common to all, or even a majority of AIEC was identified. Previously, genes encoding polyethylene glycol utilization and iron acquisition were reported to be overrepresented in AIEC relative to nonpathogenic *E. coli* [32].

In the present paper we report whole-genome sequences of 28 *E. coli* isolates from the ileum and feces of ten CD patients. The comparative analysis of these genomes and previously published strains revealed their high phylogenetic diversity as a group, high homogeneity within a single inflamed intestine, and specific genome features.

## Methods

### Patient selection

Patients were selected from two clinical centers (Central Scientific Institute of Gastroenterology and State Scientific Center of Coloproctology) in Moscow, Russian Federation, from 2012 to 2014.

Ten patients (seven males and three females, 23–47 years old, mean age 33, who met the eligibility criteria were enrolled in the study (Table 1). The inclusion criteria were the following: age above 18, endoscopically and radiologically diagnosed, and histologically confirmed Crohn’s disease. The exclusion criteria were signs of indeterminate colitis, infectious diseases, anamnesis of total colectomy, presence of stoma, and recent antibiotic treatment.

**Table 1:** Samples and patients

patient №	sex	age	disease			isolate name and origin			number of isolates	clinic	assembly
			localisation	clinical activity	endoscopic activity	biopsy	aspirate from ileum lumen	feces			
1	M	33	ileitis	low	10	RCE01–01 RCE01–02 RCE01–03 (ileum)	RCE01–04 RCE01–05	RCE01–06	6	C	RCE01
2	M	23	ileocolitis	low	13		RCE02–01 RCE02–02 RCE02–03		3	C	RCE02
3	F	37	ileocolitis	medium	14	RCE03–01 RCE03–02 RCE03–03 (ileum, caecum, sigmoid)			3	C	RCE03
4	F	40	ileocolitis-perianal	high	0	RCE04–01 (caecum)	RCE04–02 RCE04–03 RCE04–04 RCE04–05 RCE04–06		6	C	RCE04
5	M	32	ileitis-jejunitis	high	9			RCE05	1	C	RCE05
6	M	47	ileocolitis	low	15		RCE06–01 RCE06–02 RCE06–03 RCE06–04 RCE06–05		5	C	RCE06
7	M	32	ileocolitis	remission	3		RCE07		1	C	RCE07
8	F	29	ileocolitis-perianal	low	6		RCE08		1	S	RCE08
9	M	25	colitis	low	5		RCE10		1	S	RCE10
10	M	29	ileocolitis	high	8		RCE11		1	C	RCE11
Total isolates number									28		

C - Central Scientific Institute of Gastroenterology, Moscow

S - State Scientific Center of Coloproctology, Moscow

### Diagnosis and treatment

Duration of the disease was from four months to eight years. Two patients had acute disease (less than six months), eight patients had chronically relapsing disease. All patients had the confirmed Crohn’s disease three months before enrolment or earlier. Seven patients had ileocolitis (L3), two of them with perianal disease, two patients had ileitis (L1), and one patient had colitis (L2) [33]. At the enrolement, three patients had clinically severe disease (Crohn’s Disease Activity Index, CDAI > 450), one patient had moderate disease (CDAI = 320), five – mild disease (CDAI 150–220), and one patient was in clinical remission (CDAI = 110) [34]. Most patients received immunosuppressive therapy, five of them with infliximab. Two patients received steroids. None of the patients received antibiotics at the moment of enrolment in this study and two months prior to it.

### Study procedures

Three types of samples were collected for the purpose of this study. Fecal samples were collected prior to preparation for endoscopy. Bowel preparation was performed with polyethylenglycol solution. Patients underwent ileocolonoscopy at clinical centers. During this procedure samples of two types were collected, ileum liquid content was aspirated from the ileum, mucosa biopsy was taken by sterile biopsy forceps from the ileum, caecum and sigmoid (inflamed tissue near ulcers).

### Strains and cell culture

Isolation of *E. coli* was performed as follows: liquid aspirates were diluted approximately ×10^6^ fold with sterile PBS (phosphate saline buffer). Approximately 0.05 ml volume of feces were placed into 0.5 ml of sterile PBS, vortexed to homogeneity, an aliquot was diluted approximately ×10^6^ fold. Biopsy samples were vortexed in 0.2 ml of sterile PBS. For all samples, 0.1 ml of the resulting liquid was spread onto the Luria-Bertani agar plates. After overnight incubation on 37 °C, isolated colonies were identified with the Matrix Assisted Laser Desorbtion/Ionization (MALDI) Biotyper software (Bruker Daltonics, Germany) using the Microflex LT mass spectrometer (Bruker Daltonics, Germany). For DNA extraction, all *E. coli* strains were grown in the Luria-Bertani broth at 37 °C with shaking (200 RPM) overnight and collected by centrifugation. Samples and corresponding *E. coli* isolates are listed in Table 1.

The testing of susceptibility to ampicillin/sulbactam, ceftriaxone, cefotaxime, ceftazidime, cefepime, imipenem, meropenem, gentamicin, levofloxacin, and ciprofloxacin (all from Bio-Rad, USA) was performed by the disc-diffusion method using the Mueller-Hinton agar plates. The *E. coli* strain ATCC 25922 was used as a control. Current CLSI and EUCAST criteria were used for interpretation.

### Genome sequencing

Genomic DNA from individual cultures was extracted by the QIAamp DNA Mini Kit (Qiagen) according to the manufacturer’s protocol. Extracted DNA (100 ng for each sample) was disrupted into 200–300 bp fragments by Covaris S220 System (Covaris, Woburn, Massachusetts, USA). The barcode shotgun library was prepared by Ion Xpress™ Plus Fragment Library Kit (Life Technologies). PCR emulsion was performed by Ion PGM™ Template OT2 200 Kit (Life Technologies). DNA sequencing was performed by Ion Torrent PGM (Life Technologies) with the Ion 318 chip and Ion PGM™ Sequencing 200 Kit v2 (Life Technologies).

### Genome assembly and annotation

Genomes were assembled using Mira 4.0 with standard parameters for the Ion technology.

To correct Ion Torrent homopolymer errors, which could result in assembly errors [35] and artificial frame shifts in coding sequences (CDS), our HomoHomo tool was applied (freely available at www.github.com/paraslonic/HomoHomo). In short, the method consists of the following steps: mapping reads to an assembly; searching for positions with indel polymorphisms in the mapped reads; BLASTN [36] search of the assembly region around found positions; and selecting the sequence variant which is consistent both with the best BLAST hit and reads. This method reduces artificial indels in the assembly by the factor of about 2.5. The estimation is based on comparing assemblies of Ion Torrent reads before and after correction with reads from more accurate sequencing technologies such as Illumina, SOLID, and Sanger.

To produce meta-assemblies, reads from different colonies obtained from the same patient were assembled together and processed as described above.

The obtained genome sequences were annotated using PROKKA 1.7 [37].

The draft genomes are available in GenBank with the following accession numbers: RCE01 (JUDV00000000), RCE02 (JUDW00000000), RCE03 (JUDX00000000), RCE04 (JUDY00000000), RCE05 (JWJZ00000000), RCE06 (JWKA00000000), RCE06 (JWKA00000000), RCE07 (JWKB00000000), RCE08 (LAXB00000000), RCE10 (LAXA00000000), RCE11 (LAWZ00000000).

### Genome analysis

Several phylogenetic methods were used in order to verify the results. Two methods based on multiple alignment and maximum likelihood: assembly-free method with the use of precalculated groups of orthology (OG), method with de-novo assembly, annotation and OG construction.

### Comparison of individual colonies by an all-vs-all method

First, we utilized reference-free approach to examine relationships among the sequenced strains. Assemblies of individual colonies were used as a reference. All-vs-all mapping was done with the bowtie2 tool. SNPs (single nucleotide polymorphisms) were calculated with the samtools mpileup tool [38] and filtered using vcftools [39] with a *p*-value threshold of 10^−5^, 90% frequency threshold, and minimum coverage of four reads. The distance between samples was calculated as the SNP count divided by the length of the referenсe (the total length of all nucleotides with at least 4× genome coverage). The Neighbour Joining tree was build using the distance matrix by the ape package for R [40]. Scripts are available at Github (https://github.com/paraslonic/Rakitina_etal_Crohn_paper/tree/master/snpSimilarity) [41].

### Phylogenetic analysis by an assembly-free method

To assign the sequenced isolates to *E. coli* phylogroups, orthology groups (OGs) from 32 phylogenetically diverse *E. coli* and *Shigella* strains were taken from [42]. Alignments of proteins within each universal group (OGs with single-copy genes present in all analyzed genomes) were produced with ClustalW version 2.1 [43]. Consensus sequences were generated from the resulting alignments with the EMBOSS package ver. 6.6.0 [44]. These consensuses were then used as a reference for read mapping with bowtie2 ver. 2.1.0 [45]. The reads from each isolate were mapped individually, and the consensus for each OG for a particular isolate was generated with samtools [32]. The resulting consensus sequences were added to the OGs, and the groups were realigned. Alignments of all universal OGs were concatenated, all columns with gaps were removed, and the final alignment was used to construct a phylogenetic tree with PhyML v. 3.0 [46] (with 100 bootstrap replicas, the tlr optimization parameter). Previously sequenced CD-associated strains, uropathogenic (UPEC) strain JJ1886, *E. albertii* strain KF1 (GeneBank ID: CP007025), and *E. fergusonii* strain ECD227 (GeneBank ID:CM001142) were added in a similar manner, but instead of reads, nucleotide sequences of genes were used.

### Phylogenetic analysis based on assemblies and de-novo OG construction

Additionally, we evaluated whether *E. coli* isolated in the present study arose from the strains with similar lifestyles, e.g. commensal or pathogenic. For this purpose a larger ML phylogenetic tree was built without bootstraps. *E. coli* genome sequences obtained in our experiments were compared with all available complete and some unfinished *E. coli* genomes from GenBank. Only unfinished genomes that were top BLASTN hits for each CD-associated isolate were selected (the complete list is in Additional file 1). All selected genomes were assigned to one of the following groups: *Crohn*, genomes sequenced in this study; *CrohnLit*, publicly available genomes associated with CD [27–30], *Non-pathogenic*, commensal and laboratory cultivated non-pathogenic strains; *Pathogenic*, strains associated with diseases other than CD; and *Other,* with no reliable phenotype information. To avoid artificial differences resulting from different annotation pipelines, genomes from GenBank were reannotated with PROKKA 1.7 [37]. OGs were obtained using the OrthoFinder software [47, 48] with default parameters. Universal groups were selected and OGs with large gene length variation (more than 80% of the median length) were filtered out. Nucleotide sequences of genes from selected OGs were aligned by ClustalW [39]. Aligned sequences were concatenated by strain, and a Maximum Likelihood (ML) tree was built using the dnaml tool from the Emboss package [44]. All scripts for tree construction from OGs are available at GitHub https://github.com/paraslonic/Rakitina_etal_Crohn_paper/tree/master/phylogeny [41].

Multilocus sequence typing (MLST) characterizes isolates of microbial species using the DNA sequences of internal fragments of multiple housekeeping genes [49]. A MLST group was assigned by web service: mlst.warwick.ac.uk/mlst/dbs/Ecoli.

### Comparison of the gene and domain content

Principal component analysis of PFAM domains and families was performed in order to find Crohn-enriched genes and domains. For domain and domain-family annotation we applied the pfam_scan.pl v. 1.5 script [50] to annotated proteins from all strains. Annotation results were combined and binarized. For each strain a pandomain profile was obtained, defined as the vector of presence/absence values attributed to each studied genome. The length of this vector is the number of domains present in at least one strain. Bray-Curtis similarities were calculated and used to build multidimensional scaling (MDS) plot with custom R script available at GitHub [41] https://github.?>com/paraslonic/Rakitina_etal_Crohn_paper/tree/master/pfamProfiles.

To identify over- or under-represented OGs in certain groups of strains, the two-way Fisher test was used separately for each domain or OG. The comparison of the *Crohn* group with the *Commensal* group was performed. The *Crohn* group contained 10 assemblies from 10 patients involved in the current study (multiple genomes from one patient assembled together), and 17 previously published genomes [27–31]. The *Commensal* group included only strains isolated from healthy individuals [51–54] (Additional file 1 (B)). For the OG content analysis, *E. coli* genomes were reannotated. The Holm method was applied to adjust for multiple comparisons [55]. The retention index which is an indicator of consistency between a feature (i.e. the OG composition) and a tree was calculated for each domain based on the large ML tree (see Phylogenetic analysis based on assemblies and de-novo OG construction) using the phangorn package for R. Functions to OGs and domains were assigned using PFAM, Uniprot, and KEGG databases [50, 56, 57].

### Detection of plasmids

The contig was considered as a candidate plasmid, if it had no links with any other contigs of the same assembly (no reads were mapped both to an edge of this contig and to an edge of another contig), and its coverage was at least twice as high as the average coverage of the genome. All candidate contigs were then aligned with blastn against a database containing the results of the query “plasmid[title]” from NCBI nucleotide database Contigs with at least 80% nucleotide identity and 75% length coverage of a reference plasmid sequence were considered as potential plasmid contigs.

Candidate plasmid contigs were realigned with plasmid sequences with Mauve 2.4.0 [58] and visualized with genoplotR R package. In addition, the presence of a particular plasmid was identified by read mapping. Reads from each of 28 isolates left after mapping on universal OGs were then mapped with bowtie2 ver2.1.0 (local alignments) to the studied plasmids: plLF82 (NC_011917) and pJJ1886_1–5 (NC_022661, NC_022649, NC_022662, NC_022650, NC_022651). The per-nucleotide coverage was extracted with bedtools ver. 2.18.2.

### Bacteriocin production test

CDEC strains were tested for bacteriocin production by the method from [59] with minor modifications. Bacterial cells were used for inoculation of liquid TY medium containing tryptone 8 g/L, yeast extract 5 g/L, and sodium chloride 5 g/L. The 1.5% TY agar plates were subsequently inoculated by a needle stab with fresh broth cultures and the plates were incubated at 37 °C for 48 h. Bacteria were killed using chloroform vapours for 10 min. Each plate was overlaid with a 5 ml of a warm soft agar (0.7% TY agar, *w*/*v*) containing 10^7^ cells/mL of an indicator strain (K12 or MG1655). The plates were then incubated at 37 °C overnight. The assessment of bacteriocin production was based on the diameter and intensity of growth inhibition or lysis zone. The indicator strains were obtained from an in-house collection of strains. Five minutes ultraviolet-C irradiation was used as an inductor of bacteriocin expression.

The intensity of inhibition was evaluated as “strong” (a clear lysis zone), or “weak” (an opaque zone, indicating some growth inhibition).

### Phages resistance test

*E. coli* strains were tested for phage resistance (virulent, temperate, *Salmonella*-specific and male-specific) by the cross-streak and spot-test methods as described in [60]. All phages were taken from the collection of the Laboratory of Bacterial Genetics (Gamaleya Institute for Epidemiology and Microbiology).

## Results

### *E. coli* Strains cultivated from an inflamed intestine of a CD patient are closely related

**Fig. 1:**
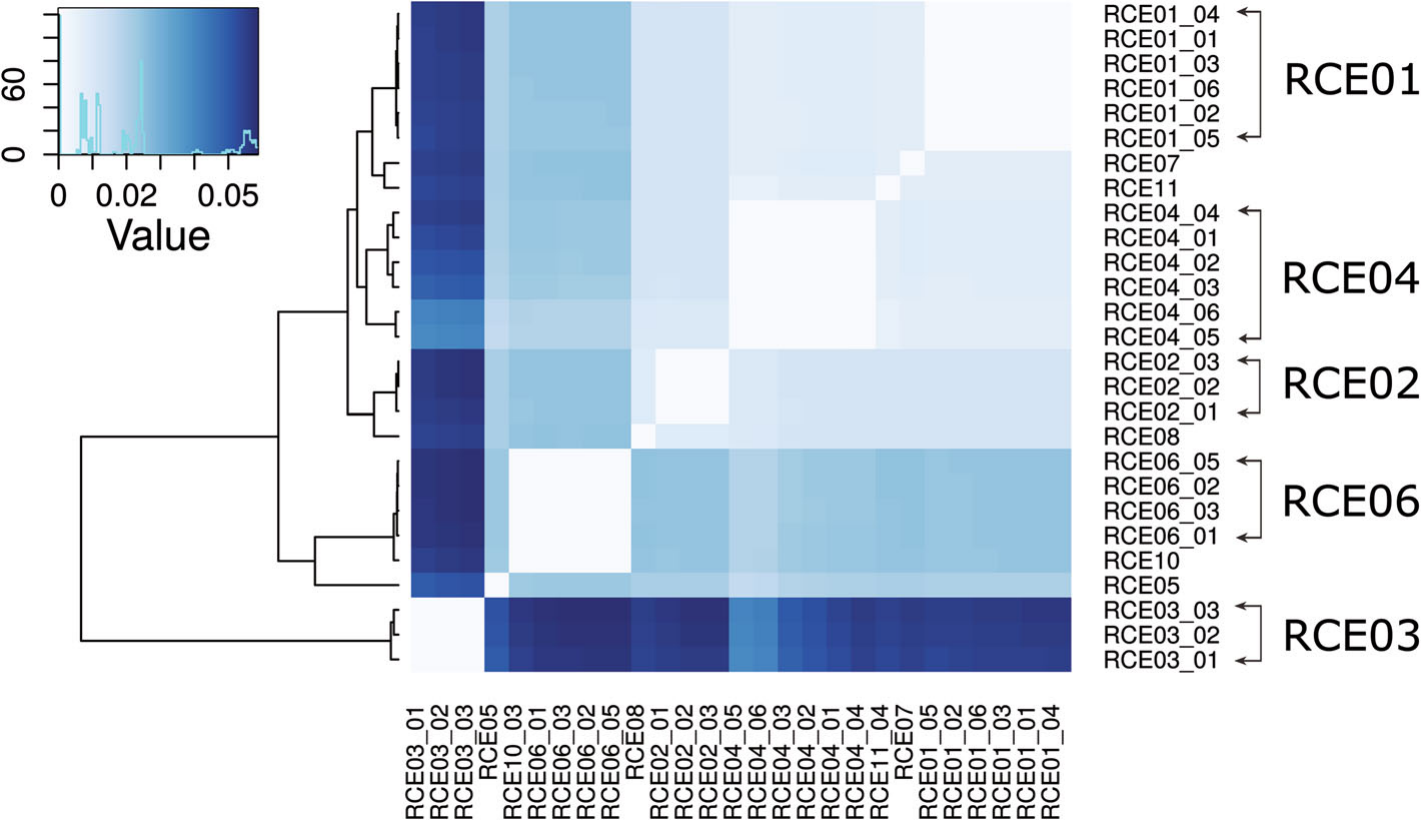
Genomic similarity of *E. coli* from individual colonies. Heatmap colors represent the number of SNPs per nucleotide (all-vs-all method). Lighter colors mean higher sequence similarity

Genome assemblies were obtained for 28 *E. coli* isolates from 10 Crohn’s disease patients (Table 1). SNP analysis of these genomic sequences (all-vs-all method) revealed that bacteria isolated from one patient tend to cluster together, even when these bacteria are isolated from different parts of the intenstine, such as in the case of patients RCE01, RCE03, and RCE04 (Fig. 1, Table 1, Additional files 2 and 3). The number of SNPs within a patient was negligible (less than 200) as compared to the interpatient diversity (on average more than 28,000). Alignment of genome sequences from different intestine parts of one patient revealed some deletions (usually a deletion of one of the smaller contigs, probably a plasmid), and minor heterogeneity (Mauve analysis, Additional file 4A). This suggested that the whole inflamed intestine of a CD patient is colonized by a single strain of *E. coli*. Basing on this conclusion we were able to merge *E. coli* genomes from each patient into a meta-assembly, and use the latter to compare strains from different patients.

### CDEC is a polyphyletic group

**Fig. 2:**
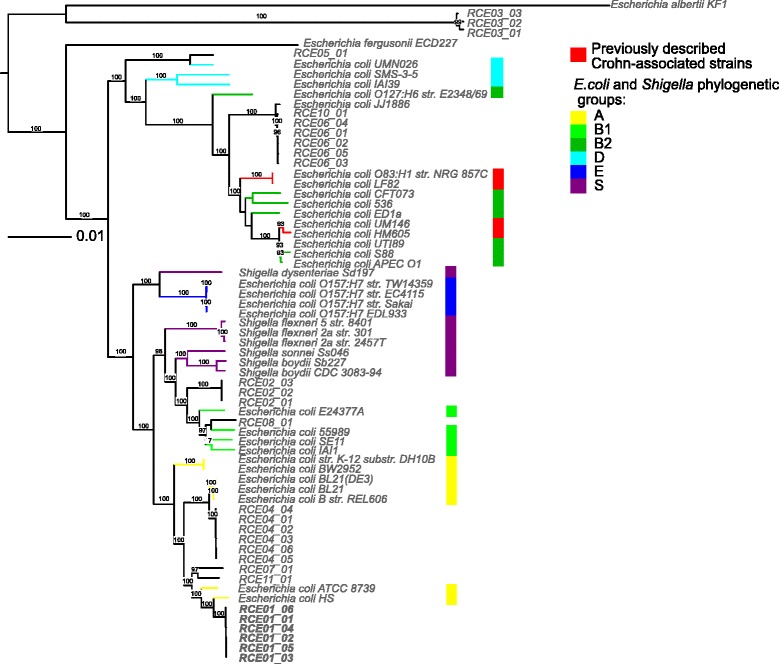
Phylogenetic analysis of *E. coli* strains. The phylogenetic tree of all universal single-copy genes was constructed by the maximum-likelihood algorithm with 100 bootstrap replicates for *E. coli* isolates from ten patients (this study), 32 *E. coli* and *Shigella* strains from [42] from phylogroups A (*yellow*), B1(*light green*), B2 (*green*), D(*cyan*), E (*blue*), S (*violet*), previously published [27–30] CD-associated strains (*red*), and uropathogenic strain JJ1886. *Escherichia albertii* KF1 and *Escherichia fergusonii* ECD227 were used as outgroups

**Fig. 3:**
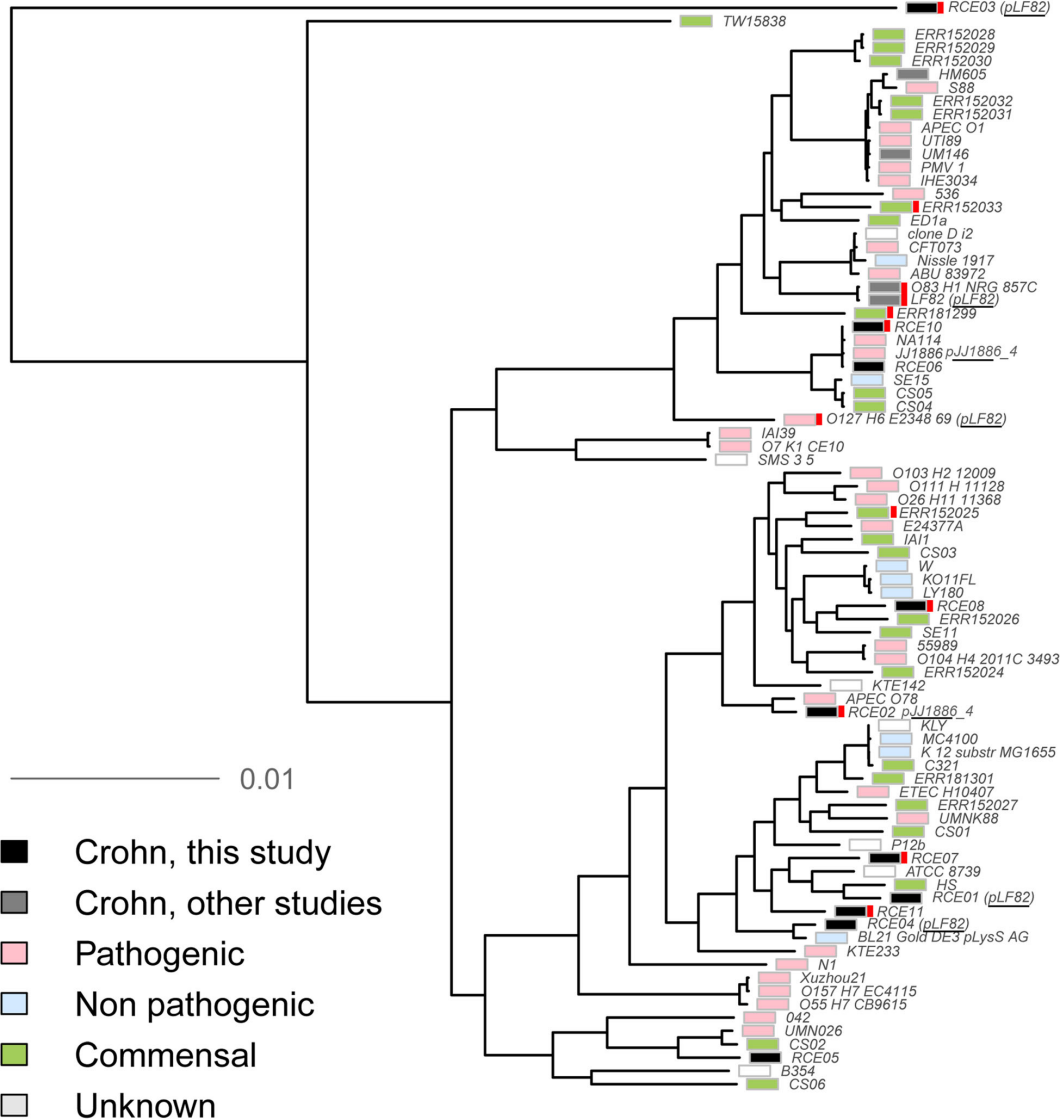
Genomic comparison of 14 CD-associated strains with pathogenic and non-pathogenic *E. coli* genomes. Maximum likelihood unrooted tree is based on the core genes. Strains from this study are colored *black* (here and on the images below indicated as Crohn); previously published CDEC (here and on the images below indicated as CrohnLit) are grey; nonpathogenic, *green*; pathogenic, *pink*; strains of undetermined pathogenicity, *white*. Derivatives of one laboratory strain are merged. Strains containing a plasmid homologous to plLF82 and pJJ1886 are indicated. *Pdu* operons from LF82 are marked with *red*

The phylogenetic analysis by two methods (SNP analysis of de novo genome assemblies and alignment of concatenated conserved proteins from 653 universal orthologous groups) shows that isolates from different patients fall into different phylogroups of *E. coli* (Figs. 2 and 3, Additional file 4B). *E. coli* from patients RCE04, RCE07, RCE11, and RCE01 belong to phylogroup A, the RCE02 isolate to phylogroup B1, the RCE05 isolate is close to phylogroup D, while RCE06 and RCE10 are placed in phylogroup B2 along with previously published genomes of CDEC strains. Isolates from patient RCE03 were shown to be more distant from *E. coli* BL21 than *E. fergusonii*. However, similarly to all *E. coli* it was resistant to lphi7S1, a phage, to which all *S. typhimurium* are susceptible and all *E. coli* are resistant (see below Additional file 5). The set of RCE03 genes was similar to other *E. coli* (see below the comparison of orthologous gene groups). The disease symptoms and clinical course of the patient RCE03 were also quite typical for CD (Additional file 2).

Hence CDEC do not form a single phylogenetic group sharing a common ancestry. The same conclusion could be drawn from the MLST typing (Additional file 6). The CD-associated strains from ten patients sequenced here fall into nine different STs including one unknown (RCE04 genome). Only ST131 has two representatives (RCE06, RCE10). This ST has been described as the fastest spreading among the B2 group [61].

At the same time, some CDEC isolates from independent sources are very similar (Figs. 2 and 3). Two classic AIEC strains from France (LF82) and Germany (O83:H1) share more than 99% sequence identity (chromosome coverage 98%) [27, 28]. Here, we revealed the high level of identity by the BLASTn alignment of chromosome sequences of two strains (patients RCE06 and RCE10) isolated in different clinics - 99% sequence identity at 94% of chromosome coverage. Weaker but pronounced similarity is observed for strains RCE07 - RCE11 (99% sequence identity at 92% of chromosome coverage). Notably, eight of the ten examined CDEC strains appeared to be phylogenetically closest to pathogenic *E. coli* (Additional file 2).

### CDEC genomes contain plasmids from pathogenic strains

Bacterial plasmids often carry genes associated with pathogenicity. To search for candidate plasmid contigs we analyzed individual CDEC isolates independently. In 24 assemblies from one to three plasmids of various length (5–100 kb) and origin were detected. Some of them had plasmids of pathogenic bacteria, e.g. uropathogenic *E. coli* and *Salmonella*, as the closest homologs, but most had high sequence identity (more than 80% coverage, 95–99% similarity) with genomes of commensal *E. coli* isolated from healthy individuals (Additional file 2). Two plasmids were found to be specific for CDEC strains.

**Fig. 4:**
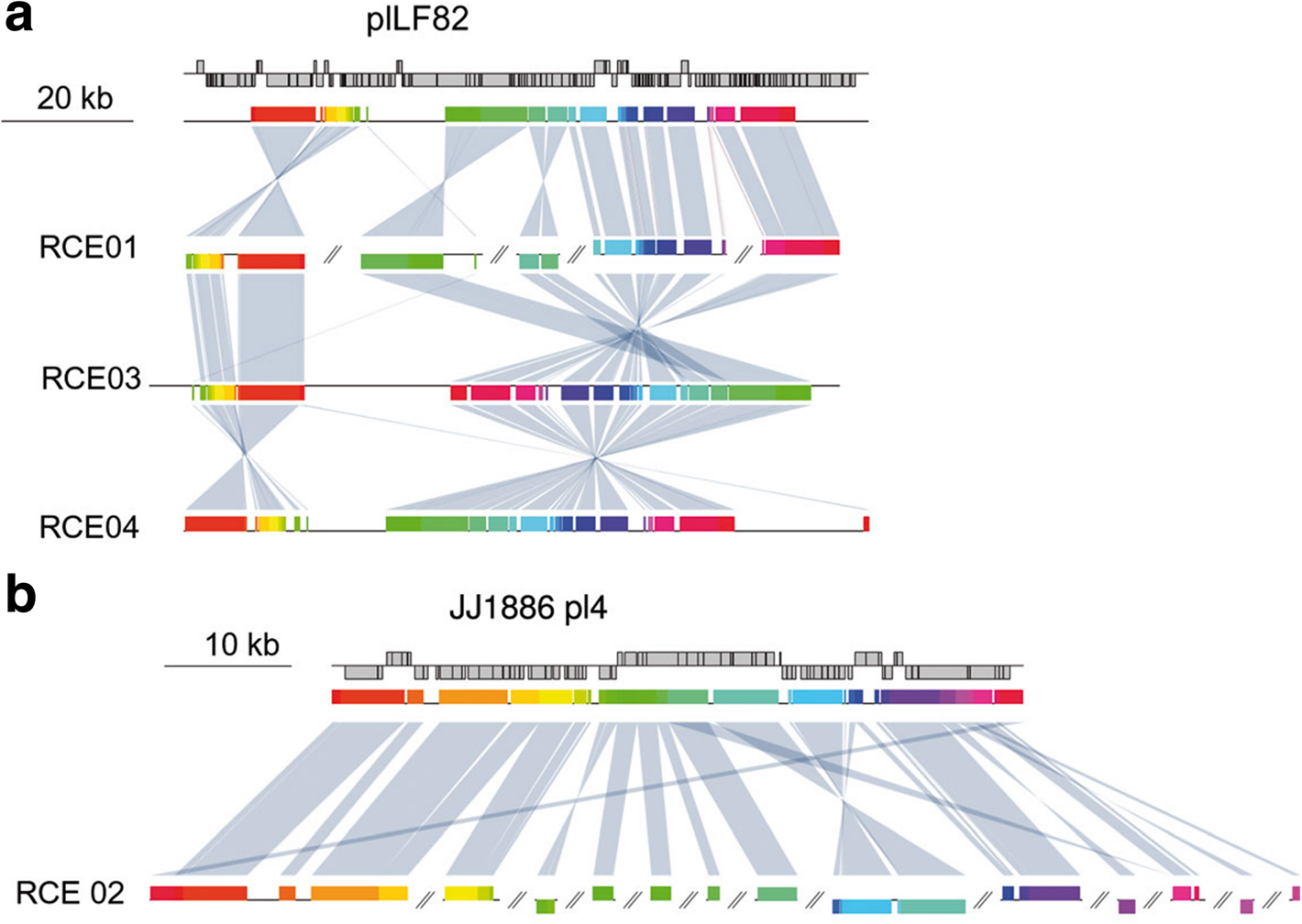
Full-length alignment of plasmids shared by CDEC strains from the present study: *E. coli* LF82 plasmid (**a**), and JJ1886 plasmid 4 (**b**). The first row in each case represents the plasmid map; other rows show homologous regions and rearrangments (MAUVE 2.4.0, default parameters) between the plasmid of interest and meta-assemblies for specific patients. Each homologous region is shown by a specific color

Plasmid contigs identified in isolates from three patients (RCE01, RCE02, RCE04) (Fig. 4a) were highly similar to the previously published reference AIEC strain LF82 [28] (Additional files 2 and 7A). Regions of plLF82 that are common for all meta-assemblies contain 99 CDS. The latter are mostly represented by phage proteins, proteins involved in DNA maintenance and conjugation, and possible virulence determinants such as enterotoxins, outer membrane proteins, resistance proteins, etc. (Additional file 7B).

A candidate plasmid detected in isolates from patient RCE02 and three previously published CDEC genomes [31] were found to be similar to plasmid pJJ1886_4 from the fatal urosepsis *E. coli* isolate JJ1886 (Fig. 4b, Additional files 2 and 7A). Genomes of two other isolates (RCE06 and RCE10) were closely related to the genome of the JJ1886 strain [30, 62]. Predicted functions of proteins shared by the CD isolates and the JJ1886 UPEC plasmid are plasmid DNA maintenance, type IV secretion, and resistance (Additional files 2 and 7C).

Plasmids plLF82 and pJJ1886_4 have no homologs in commensal or non-pathogenic *E. coli* (Additional file 7D). Plasmids with considerable similarity exist in *Yersinia pestis* (plLF82), multidrug-resistant *E. coli* from hospitals (pJJ1886_4), *Salmonella enterica*, and *Klebsiella pneumoniae* (both) (Additional file 7E, F). plLF82 has been suggested to be acquired by *E. coli* via horizontal gene transfer from *Yersinia* or *Salmonella* [28].

No sequence similarity was observed between plasmids plLF82 and pJJ1886_4, but functional analysis revealed some common functions, such as plasmid DNA maintenance and conjugation, and a few enterotoxins, outer membrane proteins, and multidrug-resistance proteins (Additional file 7B,C).

### There are no domains or genes found only in CDEC genomes

**Fig. 5:**
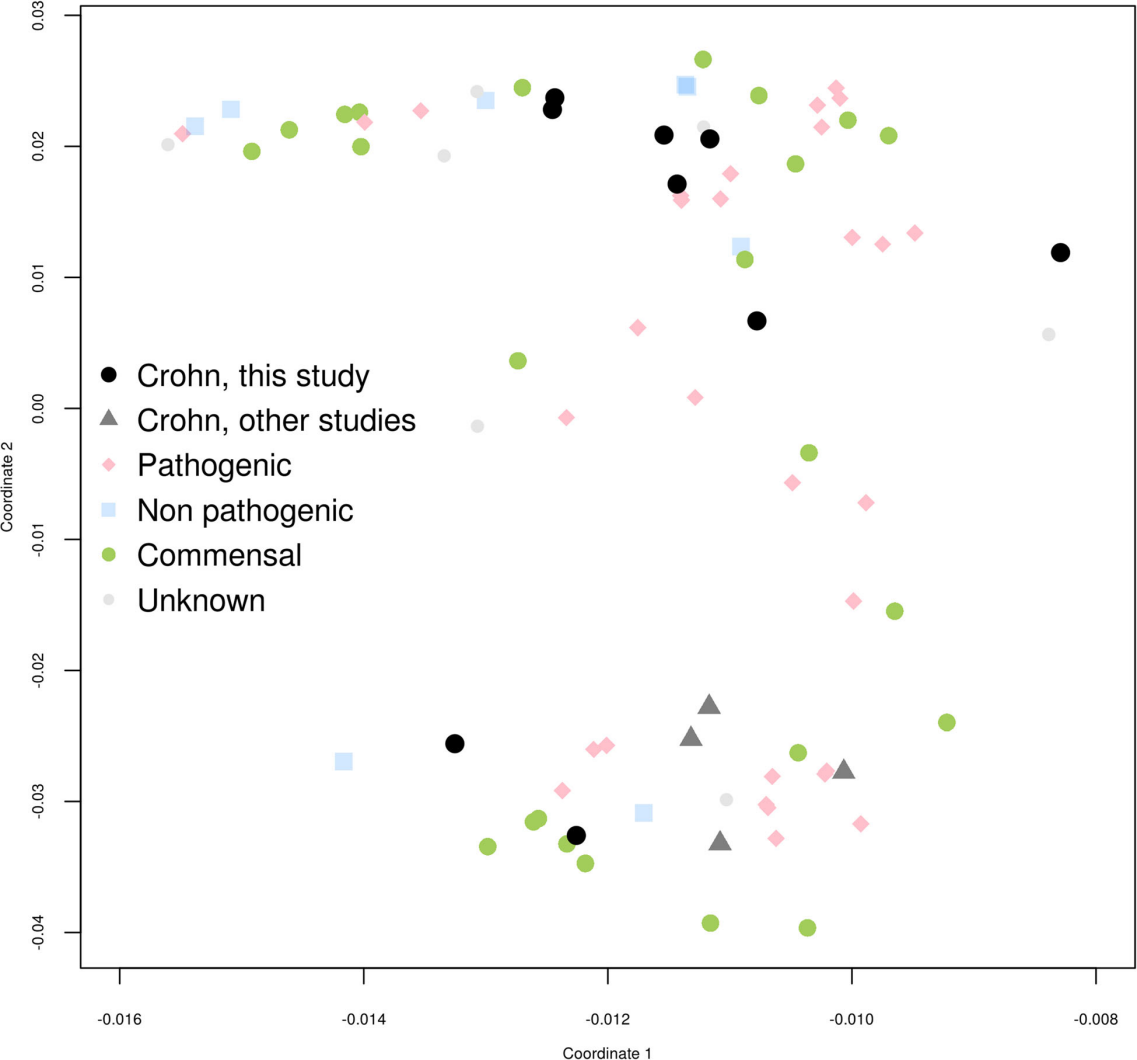
Multidimensional scaling plot of distances between the PFAM-domain content in CDEC, pathogenic, non pathogenic, and commensal *E. coli*. The colors of strains are as in Fig. 3

In order to identify potential virulence domains, we compared CDEC with other pathogenic and non-pathogenic *E. coli*. The principal component analysis of PFAM domains and families shows that CDEC do not cluster together (Fig. 5), and are scattered among pathogenic and nonpathogenic strains. Thus, CDEC as a group can not be attributed to pathogenic or non-pathogenic *E. coli* on the basis of their pandomain profile.

In order to identify genes that could influence CDEC virulence, we compared the protein composition of CDEC (27 genomes) with commensal *E. coli* strains isolated from healthy individuals (24 genomes) [29, 30, 51–54]. The complete list of strains is given in Additional file 1. In total, 143 orthologous groups (OGs) are overrepresented in the CDEC group, and 237 OGs are underrepresented (Fisher test *p*-value ≤0.05, Additional file 8). No difference was significant after adjustment for multiple testing (the Holm correction), even for those that were 10 times more often in CDEC genomes (Additional file 8). That can be partially explained by a small size of the analyzed *E. coli* dataset (51) compared to the number of regarded OGs (11,886 OGs). Hereinafter, those OGs are referred to as enriched in CDEC or commensal *E. coli* genomes.

### OGs enriched in CDEC genomes tend to fom operons

**Fig. 6:**
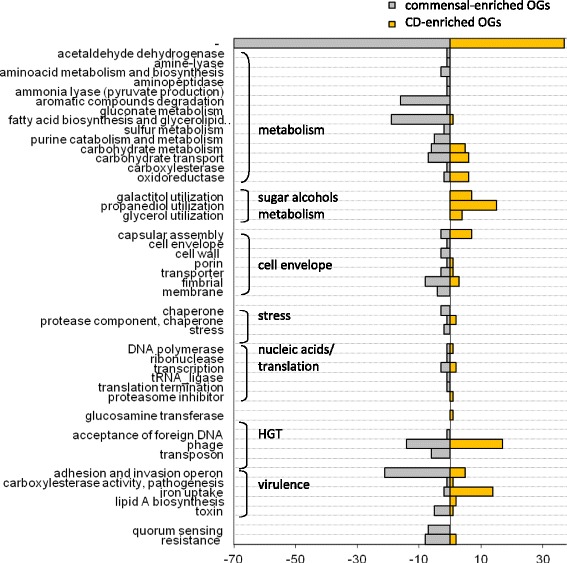
Functions of overrepresented OGs (Fisher’s test *p*-value <0.05 prior to Holm’s correction). The number of overrepresented OGs with a given function is shown on the horizontal axis for commensal strains (*grey*, *left panel*) and CDEC (*yellow*, *right panel*)

**Fig. 7:**
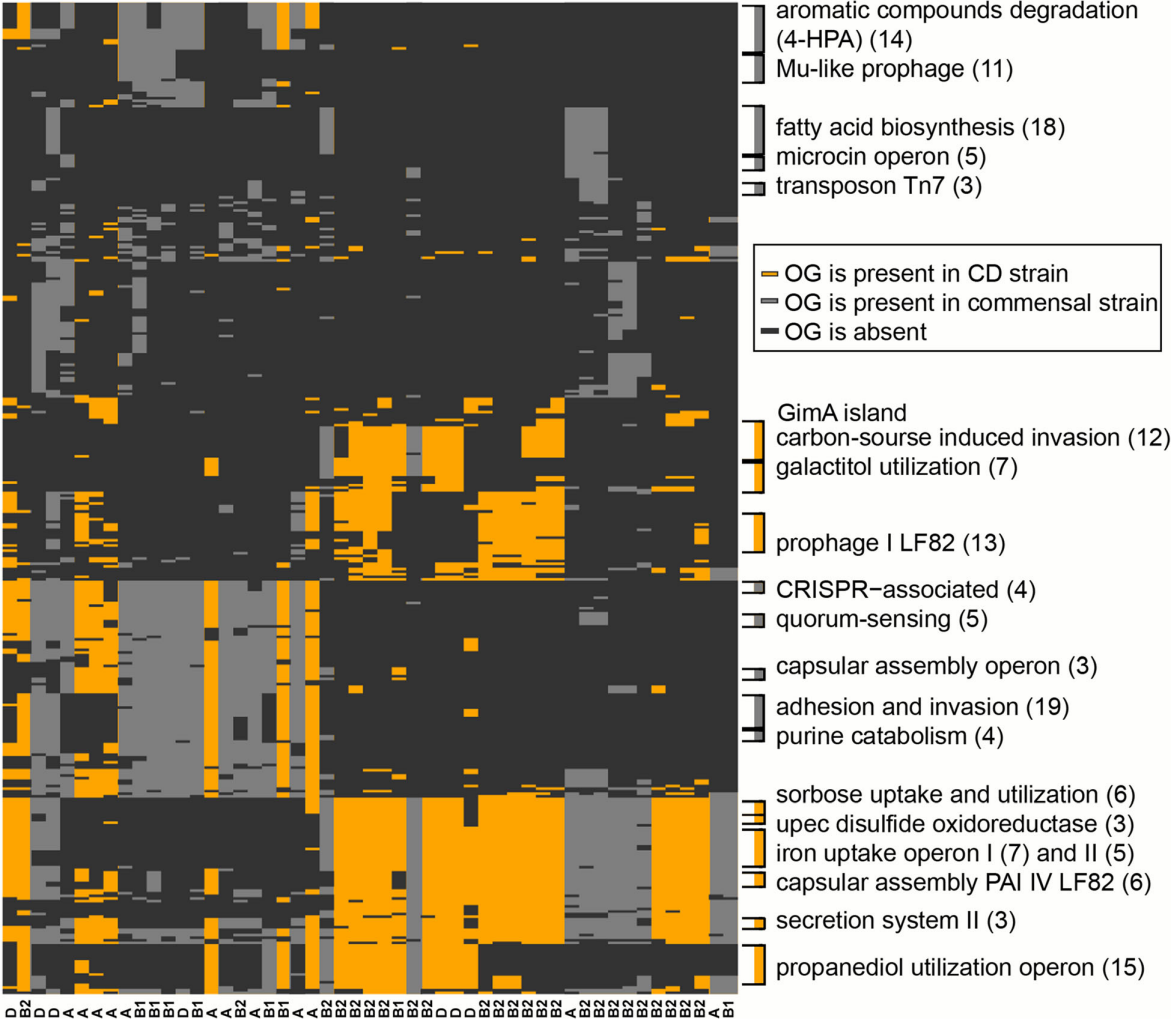
Operons and gene groups enriched in CDEC (*yellow*) and commensal *E. coli* (*grey*) (Fisher test *p*-value <0.05 prior to Holm’s correction). OGs form horisontal rows, strains – vertical columns. Genomes with similar OG patterns were clustered together using a custom R script (see Methods). that can be achieved at GitHub repository https://github.com/paraslonic/Rakitina_etal_Crohn_paper/tree/master/ogEnrichment [41]. Phylogroups of strains are indicated (A, B2, B1, D)

Most genes from CDEC-enriched or commensal-enriched OGs are located on the chromosome, and moreover, 156 of them form operons with certain finctions (Figs. 6 and 7). In the reference CDEC strain LF82 all CD-enriched operons are present, while the commensal-enriched operons are not. Six operons from LF82, namely glyoxilate metabolism - gcx part of ptn-cgl-gcx-ibe operon, capsular assembly PAI IV LF82, iron uptake operon I, sorbose uptake and utilization, prophage I LF82, and propanediol utilization operon, showed the number of enriched OGs above random probability level (Additional file 9, Fig. 7), therefore their enrichment in CDEC genomes is valid.. Genes of CD-specific plasmids did not pass Fisher’s test.

OGs overrepresented in CDEC are involved in metabolism, horizontal gene transfer (HGT), and virulence (Fig. 6).

OGs with functions associated with metabolism are mainly enriched in commensal strains (aromatic compounds degradation, fatty acid biosynthesis, and glycerolipid metabolism). The only metabolic function of CDEC-enriched OGs was utilization of sugar alcohols (propanediol, galactitol, glycerol). This function in CDEC is represented by the propanediol (15 genes) and galactiol (7 genes) utilization operons (Fig. 7).

Enrichment in OGs associated with HGT was previously reported to be characterictic of pathogenic strains leading to accumulation of pathogenic genes [49]. In our comparison, however, OGs with such HGT functions as “transposases” (transposon proteins), and “foreign DNA transfer” were enriched in commensal strains (Fig. 6). OGs with function of “foreign DNA resistance” were also enriched in the commensal group, due to the *CRISPR-*Cas locus (Figs. 6 and 7). The maindifference observed in HGT category is a presence of distinct prophages: Mu-like prophages tend to occur in commensal strains, while lambda-like prophage I from LF82 is specific for CDEC (Figs. 6 and 7). However, the phage resistance test of CDEC revealed that the presence or absence of a particular prophage in a genome cannot be directly interpreted as the evidence of the strain sensitivity (or resistance) to this phage (Additional file 5).

OGs involved in carbohydrates metabolism and uptake have equal amount of representatives among CD-enriched and commensal-enriched OGs (Fig. 6). Most of the CD-enriched OGs are rather involved in invasion, than metabolism (see below).

OGs responsible for adhesion-invasion are more common in commensal *E. coli* (Fig. 6), and are represented by the type III secretion system locus. At the same time, in CDEC this function is represented by the GimA island, containing three carbohydrate and glycerol metabolism operons (*ptn*, *cgl* and *gcx*) and one invasion *ibe* operon (Fig. 7). This island was first identified in meningitis-causing *E. coli* and proved to be responsible for carbon-source induced invasion of the blood-brain barrier [63].

In the commensal group enriched OGs asocciated with toxins are represented by the microcin operon, while in the CD group – by one gene from type II toxin-antitoxin system.

Other potentially pathogenic functions enriched in CDEC are iron uptake (Fig. 6), presented by *chu* operon and *enterobactin* gene clusters (iron uptake operons I and II, Fig. 7), and lipid A biosynthesis (3 separate OGs).

OGs encoding membrane, fimbrial proteins, transporters and those involved in cell wall/envelope assembly, are present in both commensal-enriched and CD-enriched groups (Fig. 6).

### CDEC are resistant to varying antibiotics

The antibiotic susceptibility test confirmed interpatient heterogeneity (Additional file 10). All tested *E. coli* strains expressed different phenotypes. Isolates recovered from patients RCE01 and RCE03 were pan-susceptible. Isolates from three other patients (RCE04, RCE05 and RCE06) were resistant to three or more antibiotics, thus being multidrug-resistant. All studied isolates were susceptible to ampicillin and carbapenems (imipenem and meropenem).

### CDEC produce bacteriocins, inhibiting the growth of other *E. coli* strains

Four CDEC strains (isolates from RCE04, RCE06, RCE10 and RCE11) were tested for the bacteriocin production by the method of Kohoutova [59] with slight modifications. All strains showed bactericidal effects on indicators (Additional file 11). RCE04 had the weakest bactericidal ability, showing only a weak effect on the most susceptible indicator. At that, the RCE04 strain was isolated from the ileal lumen and caecal biopsy (six isolates altogether) of one patient, suggesting it colonised the whole intestine. One may conclude that either bacteriocins are not necessary for *E. coli* to dominate the intestine, or that the expression of the RCE04 bacteriocin has not been induced in the cultivation conditions.

## Discussion

Several studies have attempted to establish whether CD-affected intestine is colonized by a single or multiple strains of *E. coli*. Indeed, different strains could abide in mucosa and lumen, within lesions and in non-affected sites (reviewed in [2]). Our analysis shows that complete genome sequences obtained from a given patient have a very low SNP rate, confirming genetic homogeneity of *E. coli* within the same intestine. Even the genomes of *E. coli* from caecum biopsy, ileum lumen and feces (patient RCE01) demonstrate high similarity, indicating that all parts of the inflamed intestine are colonized by a single strain.

Since the time the CDEC group had been defined, its phylogeny had been debated. It has been suggested [24] that this group might have evolved from a common commensal ancestor, that has become pathogenic by acquisition of virulence factors via horizontal gene transfer from related pathogenic organisms (*Klebsiella, Shigella* and *Yersinia*) [28]. Because of that, recent studies concentrate mostly on the phylogroup B2 [31]. However, in other cases high heterogeneity of CDEC serotypes and MLST groups has been observed implying, that there are only functional similarities between CDEC, and no common origin [16].

The results of our study suggest a combination of the above hypotheses. Here, high interpatient heterogeneity has been demonstrated with isolated CDEC attributed to several distinct phylogroups (Fig. 2). On the other hand, some independently isolated strains (LF82 and O83:H1, RCE06 and RCE10) are highly similar and likely share a common origin. Strains with similar chromosomes may contain unrelated plasmids and vice versa. For example, chromosomes of LF82 and O83:H1 share more then 99% homology, but their plasmids have no homologous genes. On the other hand, chromosome of RCE03 is more distant from LF82 than *E. fergusonii* (Fig. 2), while the identity between their plasmids exceeds 98% at 86% coverage. This supports the hypothesis that the CD-associated phenotype could have arisen by horizontal gene transfer (plasmid or phage), possibly from non-*E. coli* bacteria.

Another question concerning *E. coli* and Crohn’s disease is whether it is a pathogen, or just a survivor. The mechanism of strain domination has to be discovered, one possibility being that it is due to a bactericidal effect on other *E. coli* strains. Indeed, all tested CDEC demonstrated some bactericidal activity. However, in co-cultivation experiments at standard conditions, CD-isolates failed to outcompete isolates from healthy individuals (Additional file 12). Another explanation of the increased abundance of CDEC in microbiota may be better fitness in the acute inflammation conditions. This hypothesis is supported by the observed proliferation of AIEC during severe ileitis in non-sterile mice, initially induced by chemicals or protists [64].

AIEC role in CD pathogenesis is supposed to be mediated by the bacterial cell surface proteins (porins, pili, membrane proteins, glycoproteins and proteins complexes with lipopolysachharides) [2]. In that regard it is interesting that many OGs overrepresented or underrepresented in CDEC have those functions. This suggests possible differences between CD- and commensal *E. coli* cells outer surface – membrane proteins repertoire and polysaccharide composition.

Our study provides evidence that CDEC as a group is closer to pathogenic *E. coli* than to commensal one. The genomes of some CDEC strains share more than 99% identity with defined pathogenic strains and/or contain plasmids closely related to those of defined pathogenic strains (Additional file 2). While no universal pathogenic feature was found in the genomes of the analyzed strains, several protein functions were more prominent in the CD-associated group of *E. coli*, and that could be relevant to the possible pathogenicity of these strains.

**Fig. 8:**
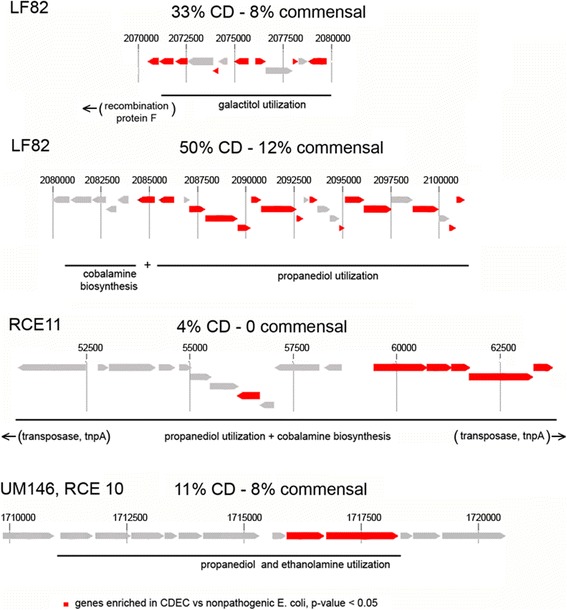
Schematic representation of the propanediol and galactitol operons in CDEC genomes. For each operon the reference strain and the percent of genomes containing it is indicated (CDEC vs commensal)

One of the functions of genes enriched in CDEC is propanediol utilization (similar results were obtained in [12]). It is interesting that the propanediol utilization operons in CDEC are of diverse origins: the operon from LF82 (O83:H1, four strains from the present study and 7 CD strains from [31]) has homologs in pathogenic *E. coli* strains, *E. albertii*, and *Shigella* sp. The operon from two other CD strains (UM146 and RCE10) is similar to the pathogeniсity island II from *E. coli* 536 strain. The operon from the RCE11 strain is similar to that of *Citrobacter* and *Klebsiella* spp. This provides additional support to the suggestion that CD-specific features in *E. coli* strains are not specific genes, but functions, probably obtained from independent sources via horizontal gene transfer (Fig. 8). Indeed, in many cases these genes form operons flanked by genes encoding transposases or recombination proteins (Fig. 8).

Recent publications show that the utilization of 1,2-propanediol is closely linked to intestinal proliferation and virulence of *Listeria monocytogenes*, enteropathogenic *E. coli* (EPEC), *Salmonella enterica* and *Enterococcus faecalis* (reviewed in [65]). Further, the genes required for 1,2-propanediol degradation are necessary for *Salmonella* replication within macrophages [66]. 1,2-propanediol utilization is important for the growth in host tissues since its precursor, fucose is found in glycoconjugates of intestinal cells involved in host-parasite interactions [67]. 1,2-propanediol can be utilized by members of *Enterobacteriaceae* via aerobic and/or anaerobic pathways [68]. A normal condition in the intestine is anaerobic, whence the aerobic pathway is much more efficient. So, the inflamed intestine would provide bacteria possessing this pathway with both an abundant substrate and the conditions for its optimal utilization. At that, *Salmonella typhimurium* has been suggested to induce acute inflammation in the intestine to provide aerobic conditions for ethanolamine utilization (a pathway close to the propanediol utilization) [69]. One could speculate that a similar mechanism forms a base for the CD pathogenesis.

Of 14 strains containing the *pdu* operon similar to that of LF82, nine are positioned close to the galactitol utilization locus (Fig. 8). These genes are functionally analogous to the *gat* operon that is common to all *E. coli,* however without any sequence similarity. The closest relatives of the CD-specific galactiol catabolism operon from LF82 are found in *Klebsiella sp. Salmonella enterica*, *Enterobacter spp.,* and *Listeria monocytogenes*. Previously, the sets of genes for the galactitol catabolism in *Enterobacteriaceae* were reported to be involved in horizontal gene transfer and recombination events [70]. It is hard to tell whether the additional galactitol operon has any specific function, but this pathway is connected with the gut colonization. For example, genes involved in the galactitol catabolism are induced in *E. coli* by growth on mucus [71] and show differential expression during biofilm formation [72]. Also, multiple mutations in those genes rapidly occur in laboratory strains of *E. coli* transferred from the minimal growth media to the mouse gut, suggesting they are under specific selective pressure in natural conditions [73].

The above observations suggest that CD-enriched genomic features of CDEC presumably provide bacteria with an increased ability for intestine colonization. These genes are organized in clusters that are likely acquired by *E. coli* from other members of *Enterobacteriaceae* via horizontal gene transfer.

Hence it seems that CDEC are not just commensal strains able to survive the acute inflammation. They have some characteristics of pathogenic *E. coli*. None of these are as straightforward as the Shiga toxin. All of them have been reported to improve colonization and to increase survival and fitness. It is possible that while persisting in the intestine, certain *E. coli* strains accumulate more and more of such improvements until taken together they may push the strain from commensality to pathogenesis.

Common CD-factors, if any, may not be specific genes or proteins, but rather functions performed by different genes in different strains. The heterogeneity of CDEC does not exclude the possibility that different groups of CDEC can possess different mechanisms for the survival in the inflamed intestine and therefore for the development of Crohn’s disease response in a patient, suggesting that specific treatment might be required in each case.

## Conclusions

Our findings suggest that CDEC are of diverse phylogeny. However, some strains isolated from independent sources possess highly similar chromosomes or plasmids. No CD-specific genes or functional domains were found to be present in all CD-associated strains. However, some genes and operons are more often found in the genomes of CDEC than in commensal *E. coli*. They are mainly linked to the gut colonization and utilization of propanediol and other sugar alcohols.

## Additional files

Additional file 1: Strains used for the comparative analysis. (A) Strains used for the phylogenetic analysis. Characteristics of non-CD strains with whole-sequenced genomes used for the comparative analysis. (B) Strains used for OG content comparison of CDEC and commensal strains. (XLS 53 kb)

Additional file 2: The genomes sheet lists assemblies, sequence quality characteristics, the closest genomes and plasmids. (XLS 35 kb)

Additional file 3: The number of SNPs between genomes of individual *E. coli* colonies. (XLS 26 kb)

Additional file 4: (A) Mauve alignment of CD-*E. coli* isolates from one patient (alignment made for RCE01, RCE02, RCE03, RCE03 and RCE06). (B) Heatmap of the phylogenetic distance between *E. coli* strains. Distances between strains are calculated as the median distance of core genes (see Methods). Phylogroups of *E. coli* are designated (A, B1, E, B2, D, F). RCE03 is not shown because of its low similarity to other *E. coli* strains. (DOC 4350 kb)

Additional file 5: Phage sensitivity test of *E. coli* CD-isolates (see Methods). (DOC 45 kb)

Additional file 6: Multi locus sequence types (MLST) were determined by the sequence of seven housekeeping genes with web server mlst.warwick.ac.uk [48]. (XLS 17 kb)

Additional file 7: plLF82 homologs in CD-associated *E. coli*. (A) Sequence similarity of CD isolates with the plasmid pLF82 (100 kb), (B) and (C) functions of proteins common between pLF82 or pJJ1886_4 and CD isolates The functions were obtained from original annotation, complemented by Prokka annotation and BLAST protein similarity search. (D) Sequence similarity of commensal *E. coli* isolates with plasmids plLF82 and pJJ1886_4 (script for the data is described in supplementary data https://github.com/paraslonic/Rakitina_etal_Crohn_paper/tree/master/plasmidCoverage [41]. (DOC 228 kb)

Additional file 8: Comparison of the OG content of CD-isolates and commensal strains. Columns: OG – orthologous group number; Function – orthologous group function; gene group / global function – function of the operon or a major pathway in which OG participates; crohnall.yes − number of genomes from CrohnAll group containing the OG; crohnall.no − number of genomes from CrohnAll group not containing the OG; commensal.yes − number of genomes from commensal group containing the OG; commensal.no − number of genomes from commensal group not containing the OG; pvalues – Fisher’s test *p*-value; pvalues.adj – *p*-value after Holm’s adjustment; CD% / commensal % = % of CD genomes containing the subject OG divided by % of commensal strains containing the subject OG. (XLS 1813 kb)

Additional file 9: Test of non randomness of the operon enrichment. (DOC 871 kb)

Additional file 10: Antibiotics susceptibility test of CD isolates (see Materials and Methods). (XLS 29 kb)

Additional file 11: Bacteriocin production test in CD-associated strains. (DOC 42 kb)

Additional file 12: Competition co-culture of CD-*E. coli* and isolates from healthy individuals. (DOC 56 kb)

## Abbreviations

AIEC: adherent-invasive *E. coli*
CD: coding sequence
CD: Crohn’s disease
CDEC: *E. coli* isolated from Crohn’s disease patients
CEACAM6: carcinoembryonic antigen related cell adhesion molecule 6
EPEC: enteropathogenic *E. coli*
IBD: inflammatory bowel disease
KEGG: Kyoto Encyclopedia of Genes and Genomes
MALDI: Matrix Assisted Laser Desorbtion/Ionization
MLST: multilocus sequence typing
NP: nonpathogenic
OG: orthology groups
OmpC: Outer membrane protein C
ORF: open reading frame
PBS: phosphate saline buffer
SNP: single nucleotide polymorphism
UPEC: uropathogenic *E. coli*

## References

[1] Chassaing B, Darfeuille-Michaud A (2011). The commensal microbiota and enteropathogens in the pathogenesis of inflammatory bowel diseases. Gastroenterology.

[2] Martinez-Medina M, Garcie-Gil LG (2014). *Escherichia coli* In chronic inflammatory bowel diseases: an update on adherent invasive *Escherichia coli* pathogenicity. World J Gastrointest Pathophysiol.

[3] Franke A, McGovern DP, Barrett JC, Wang K, Radford-Smith GL, Ahmad T, Lees CW, Balschun T, Lee J, Roberts R (2010). Genome-wide meta-analysis increases to 71 the number of confirmed Crohn’s disease susceptibility loci. Nat Genet.

[4] Conte MP, Schippa S, Zamboni I, Penta M, Chiarini F, Seganti L (2006). Gut-associated bacterial microbiota in paediatric patients with inflammatory bowel disease. Gut.

[5] Kotlowski R, Bernstein CN, Sepehri S, Krause DO (2007). High prevalence of *Escherichia coli* belonging to the B2+D phylogenetic group in inflammatory bowel disease. Gut.

[6] Martin HM, Campbell BJ, Hart CA, Mpofu C, Nayar M, Singh R (2004). Enhanced *Escherichia coli* adherence and invasion in Crohn's disease and colon cancer. Gastroenterology.

[7] Darfeuille-Michaud A, Neut C, Barnich N, Lederman E, Di Martino P, Desreumaux P (1998). Presence of adherent *Escherichia coli* strains in ileal mucosa of patients with Crohn's disease. Gastroenterology.

[8] Gevers D, Kugathasan S, Denson LA, Vazquez-Baeza Y, Van Treuren W, Ren B (2014). The treatment-naive microbiome in new-onset Crohn’s disease. Cell Host Microbe.

[9] Boudeau J, Glasser AL, Masseret E, Joly B, Darfeuille-Michaud A. Invasive ability of an *Escherichia coli* strain isolated from the ileal mucosa of a patient with Crohn’s disease. Infect Immun. 1999;67:4499–509. pmid:10456892/pmc96770. 10.1128/iai.67.9.4499-4509.1999 PMC96770 10456892

[10] Glasser A-L, Boudeau J, Barnich N, Perruchot M-H, Colombel J-F, Darfeuille-Michaud A (2001). Adherent invasive *Escherichia coli* strains from patients with Crohn's disease survive and replicate within macrophages without inducing host cell death. Infect Immun.

[11] Lapaquette P, Glasser AL, Huett A, Xavier RJ, Darfeuille-Michaud A (2010). Crohn’s disease-associated adherent-invasive *E. coli* are selectively favoured by impaired autophagy to replicate intracellularly. Cell Microbiol.

[12] Dogan B, Scherl E, Bosworth B, Yantiss R, Altier C, McDonough PL (2013). Multidrug resistance is common in *Escherichia coli* associated with Ileal Crohn’s disease. Inflamm Bowel Dis.

[13] Martinez-Medina MX, Aldeguer M, Lopez-Siles F, González-Huix C, López-Oliu G, Dahbi G (2009). molecular diversity of *Escherichia coli* in the human gut: new ecological evidence supporting the role of adherent-invasive *E. coli* (AIEC) in Crohn's disease. Inflamm Bowel Dis.

[14] Carvalho FA, Barnich N, Sivignon A, Darcha C, Chan CH, Stanners CP (2009). Crohn’s disease adherent-invasive *Escherichia coli* colonize and induce strong gut inflammation in transgenic mice expressing human CEACAM. J Exp Med.

[15] Small CL, Reid-Yu SA, McPhee JB, Coombes BK (2013). Persistent infection with Crohn’s disease-associated adherent-invasive *Escherichia coli* leads to chronic inflammation and intestinal fibrosis. Nat Commun.

[16] Rolhion N, Carvalho FA, Darfeuille-Michaud A (2007). OmpC and the sigma(E) regulatory pathway are involved in adhesion and invasion of the Crohn's disease-associated *Escherichia coli* strain LF82. Mol Microbiol.

[17] Vejborg RM, Hancock V, Petersen AM, Krogfelt KA, Klemm P (2011). Comparative genomics of *Escherichia coli* isolated from patients with inflammatory bowel disease. BMC Genomics.

[18] Baumgart M, Dogan B, Rishniw M, Weitzman G, Bosworth B, Yantiss R (2007). Culture independent analysis of ilealmucosa reveals a selective increase in invasive *Escherichia coli* of novel phylogeny relative to depletion of Clostridiales in Crohn’s disease involving the ileum. The ISME Journal.

[19] Conte MP, Longhi C, Marazzato M, Conte AL, Aleandri M, Lepanto MS (2014). Adherent-invasive *Escherichia coli* (AIEC) in pediatric Crohn’s disease patients: phenotypic and genetic pathogenic features. BMC Research Notes.

[20] Sepehri S, Khafipour E, Bernstein CN, Coombes BK, Pilar AV, Karmali M (2011). Characterization of *Escherichia coli* isolated from gut biopsies of newly diagnosed patients with inflammatory bowel disease. Inflamm Bowel Dis.

[21] Sasaki M, Sitaraman SV, Babbin BA, Gerner-Smidt P, Ribot EM, Garrett N (2007). Invasive *Escherichia coli* are a feature of Crohn’s disease. Lab Investig.

[22] Sobieszczanska B, Kasprzykowska U, Turniak M, Maciejewski H, Franiczek R, Duda-Madej A (2012). Virulence genes profiles and phylogenetic origin of *Escherichia coli* from acute and chronic intestinal diseases revealed by comparative genomic hybridization microarray. Pol J Microbiol.

[23] Sobieszczańska BA, Duda-Madej AB, Turniak MB, Franiczek R, Kasprzykowska U, Duda AK, Rzeszutko M, Iwańczak B (2012). Invasive properties, adhesion patterns and phylogroup profiles among *Escherichia coli* strains isolated from children with inflammatory bowel disease. Adv Clin Exp Med.

[24] Petersen AM, Nielsen EM, Litrup E, Brynskov J, Mirsepasi H, Krogfelt KA (2009). A phylogenetic group of *Escherichia coli* associated with active left-sided inflammatory bowel disease. BMC Microbiol.

[25] Masseret E, Boudeau J, Colombel JF, Neut C, Desreumaux P, Joly B (2001). Genetically related *Escherichia coli* strains associated with Crohn’s disease. Gut.

[26] Schippa S, Conte MP, Borrelli O, Iebba V, Aleandri M, Seganti L (2009). Dominant genotypes in mucosaassociated *Escherichia coli* strains from pediatric patients with inflammatory bowel disease. Inflamm Bowel Dis.

[27] Nash JH, Villegas A, Kropinski AM, Aguilar-Valenzuela R, Konczy P, Mascarenhas M (2010). Genome sequence of adherent-invasive Escherichia coi and comparative genomic analysis with other E coli pathotypes. BMC Genomics.

[28] Miquel S, Peyretaillade E, Claret L, de Vallée A, Dossat C, Vacherie B, Zineb el H, Segurens B, Barbe V, Sauvanet P, Neut C, Colombel JF, Medigue C, Mojica FJ, Peyret P, Bonnet R, Darfeuille-Michaud A. Complete genome sequence of Crohn's disease-associated adherent-invasive *E. coli* strain LF82. PLoS One.2010;5(9):e12714. 10.1371/journal.pone.0012714 PMC2941450 20862302

[29] Krause DO, Little AC, Dowd SE, Bernstein CN (2011). Complete genome sequence of adherent invasive *Escherichia coli* UM146 isolated from Ileal Crohn’s disease biopsy tissue. J Bacteriol.

[30] Clarke DJ, Chaudhuri RR, Martin HM, Campbell BJ, Rhodes JM, Constantinidou C (2011). Complete genome sequence of the Crohn’s disease-associated adherent-invasive *Escherichia coli* strain HM605. J Bacteriol.

[31] O'Brien CL, Bringer MA, Holt KE, Gordon DM, Dubois AL, Barnich N, Darfeuille-Michaud A, Pavli P. Comparative genomics of Crohn's disease-associated adherent-invasive *Escherichia coli*. Gut. 2016; doi:10.1136/gutjnl-2015-311059. 10.1136/gutjnl-2015-311059 27196580

[32] Dogan B, Suzuki H, Herlekar D, Sartor RB, Campbell BJ, Roberts CL (2014). Inflammation-associated adherent-invasive *Escherichia coli* are enriched in pathways for use of propanediol and iron and M-cell translocation. Inflamm Bowel Dis.

[33] Silverberg MS, Satsangi J, Ahmad T, Arnott ID, Bernstein CN, Brant SR, et al. Toward an integrated clinical, molecular and serological classification of inflammatory bowel disease: Report of a Working Party of the 2005 Montreal World Congress of Gastroenterology. Can J Gastroenterol 2005;19(SupplA):5–36. 10.1155/2005/269076 16151544

[34] Best WR, Becktel JM, Singleton JW, Kern F (1976). Development of a Crohn's disease activity index. National Cooperative Crohn's Disease Study Gastroenterology.

[35] Bragg LM, Stone G, Butler MK, Hugenholtz P, Tyson GW (2013). Shining a light on dark sequencing: characterising errors in ion torrent PGM data. PLoS Comput Biol.

[36] Altschul SF, Madden TL, Schäffer AA, Zhang J, Zhang Z, Miller W (1997). Gapped BLAST and PSIBLAST:a new generation of protein database search programs. Nucl Acids Res..

[37] Seemann T (2014). Prokka: rapid prokaryotic genome annotation. Bioinformatics.

[38] Li H, Handsaker B, Wysoker A, Fennell T, Ruan J, Homer N (2009). The sequence alignment/map format and SAMtools. Bioinformatics.

[39] Danecek P, Auton A, Abecasis G, Albers CA, Banks E, DePristo MA (2011). 1000 genomes project analysis group. Bioinformatics.

[40] Paradis E, Claude J, Strimmer K (2004). APE: analyses of phylogenetics and evolution in R language. Bioinformatics.

[41] Rakitina DV, Manolov AI, Kanygina AV, Garushyants SK, Baikova JP, Alexeev DG, Ladygina VG, Kostriukova ES, Larin AK, Semashko TA, Karpova IY, Babenko VV, Ismagilova RK, Malanin SY, Gelfand MS, Ilina EN, Gorodnichev RB, Lisitsyna ES, Aleshkin GI, Scherbakov PL, Khalif IL, Shapina MV, Maev IV, Andreev DN, Govorun VM. Data from: Genome analysis of *E. coli* isolated from Crohn’s disease patients. doi: 10.5281/zenodo.546444

[42] Gordienko EN, Kazanov MD, Gelfand MS (2013). Evolution of pan-genomes of *Escherichia coli*, Shigella spp.,and Salmonella enterica. J Bacteriol.

[43] Larkin MA, Blackshields G, Brown NP, Chenna R, McGettigan PA, McWilliam H (2007). Clustal W and Clustal X version 2.0. Bioinformatics.

[44] Rice P, Longden I, Bleasby A (2000). EMBOSS: the European molecular biology open software suite. Trends Genet.

[45] Langmead B, Salzberg SL (2012). Fast gapped-read alignment with bowtie 2. Nat Methods.

[46] Guindon S, Dufayard J-F, Lefort V, Anisimova M, Hordijk W, Gascuel O (2010). New algorithms and methods to estimate maximum-likelihood phylogenies: assessing the performance of PhyML 3.0. Syst Biol.

[47] Emms DM, Kelly S (2015). OrthoFinder: solving fundamental biases in whole genome comparisons dramatically improves orthogroup inference accuracy. Genome Biol.

[48] Quast C, Pruesse E, Yilmaz P, Gerken J, Schweer T, Yarza P (2013). The SILVA ribosomal RNA gene database project: improved data processing and web-based tools. Nucl Acids Res.

[49] Wirth T, Falush D, Lan R, Colles F, Mensa P, Wieler LH, Karch H, Reeves PR, Maiden MC, Ochman H, Achtman M (2006). Sex and virulence in *Escherichia coli*: an evolutionary perspective. MolMicrobiol.

[50] Finn RD, Bateman A, Clements J, Coggill P, Eberhardt RY, Eddy SR (2014). The Pfam protein families database. Nucl Acids Res..

[51] Luoa C, Walkc ST, Gordond DM, Feldgardene M, Tiedjef JM, Konstantinidis KT (2011). Genome sequencing of environmental *Escherichia coli* expands understanding of the ecology and speciation of the model bacterial species. PNAS.

[52] Lajoie MJ, Rovner AJ, Goodman DB, Aerni HR, Haimovich AD, Kuznetsov G, Mercer JA, Wang HH, Carr PA, Mosberg JA, Rohland N, Schultz PG, Jacobson JM, Rinehart J, Church GM, Isaacs FJ (2013). Genomically recoded organisms expand biological functions. Science.

[53] de Muinck EJ, Lagesen K, Afset JE, Didelot X, Rønningen KS, Rudi K, Stenseth NC, Trosvik P (2013). Comparisons of infant *Escherichia coli* isolates link genomic profiles with adaptation to the ecological niche. BMC Genomics.

[54] Garrett M, Parker J, Stephens CM (2014). Draft genome sequences of antibiotic-resistant commensal *Escherichia coli*. Genome Announc.

[55] Holm S (1979). A simple sequentially rejective multiple test procedure. Scand J Statist.

[56] Bairoch A, Apweiler R, Wu CH, Barker WC, Boeckmann B, Ferro S, Gasteiger E, Huang H, Lopez R, Magrane M, Martin MJ, Natale DA, O'Donovan C, Redaschi N, Yeh LS (2005). The universal protein resource (UniProt). Nucleic Acids Res.

[57] Kanehisa M, Sato Y, Kawashima M, Furumichi M, Tanabe M (2016). KEGG as a reference resource for gene and protein annotation. Nucleic Acids Res.

[58] Darling AC, Mau B, Blattner FR, Perna NT (2004). Mauve: multiple alignment of conserved genomic sequence with rearrangements. Genome Res.

[59] Kohoutova D, Smajs D, Moravkova P, Cyrany J, Moravkova M, Forstlova M (2014). *Escherichia coli* strains of phylogenetic group B2 and D and bacteriocin production are associated with advanced colorectal neoplasia. BMC Infect Dis.

[60] Aleshkin GI, Smelkova OI, Timakova NV, Dobrynina OI, Umiarov AM, Rusina OI, Rusina OI, Markov AP, Bol'shakova TN (2014). Role of phage LØ7 lysogeny in genetic variability of *Escherichia coli* [article in Russian]. Zh Mikrobiol (Moscow).

[61] Rogers B, Sidjabat H, Paterson D (2011). *Escherichia coli* O25b-ST131: a pandemic, multiresistant, community-associated strain. J Antimicrob Chemother.

[62] Andersen PS, Stegger M, Aziz M, Contente-Cuomo T, Gibbons HS, Keim P, et al. Complete Genome Sequence of the Epidemic and Highly Virulent CTX-M-15-Producing H30-Rx Subclone of *Escherichia coli* ST131. Genome Announc. 2013;doi:10.1128/genomeA.00988-13. 10.1128/genomeA.00988-13 PMC3853059 24309736

[63] Huang SH, Chen YH, Kong G, Chen SH, Besemer J, Borodovsky M, Jong A (2001). A novel genetic island of meningitic *Escherichia coli* K1 containing the ibeA invasion gene (GimA): functional annotation and carbonsource-regulated invasion of human brain microvascular endothelial cells. Funct Integr Genomics.

[64] Craven M, Egan CE, Dowd SE, McDonough SP, Dogan B, Denkers EY (2012). Inflammation drives Dysbiosis and bacterial invasion in murine models of Ileal Crohn’s disease. PLoS One.

[65] Staib L, Fuchs TM (2014). From food to cell: nutrient exploitation strategies of enteropathogens. Microbiology.

[66] Conner CP, Heithoff DM, Julio SM, Sinsheimer RL, Mahan MJ (1998). Differential patterns of acquired virulence genes distinguish salmonella strains. Proc Natl Acad Sci U S A.

[67] Klumpp J, Fuchs TM (2007). Identification of novel genes in genomic islands that contribute to salmonella typhimurium replication in macrophages. Microbiology.

[68] Toraya T, Honda S, Fukui S. Fermentation of 1,2-propanediol with 1,2-ethanediol by some genera of Enterobacteriaceae, involving coenzyme B12-dependent diol dehydratase. J Bacteriol. 1979;139:39–47. pmid:378959/pmcid:pmc216824. 10.1128/jb.139.1.39-47.1979 PMC216824 378959

[69] Thiennimitr P, Winter SE, Winter MG, Xavier MN, Tolstikov V, Huseby DL (2011). Intestinal inflammation allows salmonella to use ethanolamine to compete with the microbiota. Proc Natl Acad Sci U S A.

[70] Shakeri-Garakani A, Brinkkötter A, Schmid K, Turgut S, Lengeler JW (2004). The genes and enzymes for the catabolism of galactitol, D-tagatose, and related carbohydrates in Klebsiella oxytoca M5a1 and other enteric bacteria display convergent evolution. Mol Gen Genomics.

[71] Fabich AJ, Jones SA, Chowdhury FZ, Cernosek A, Anderson A, Smalley D, McHargue JW, Hightower GA, Smith JT, Autieri SM, Leatham MP, Lins JJ, Allen RL, Laux DC, Cohen PS, Conway T (2008). Comparison of carbon nutrition for pathogenic and commensal *Escherichia coli* strains in the mouse intestine. Infect Immun.

[72] Domka J, Lee J, Bansal T, Wood TK (2007). Temporal gene-expression in *Escherichia coli* K-12 biofilms. Environ Microbiol.

[73] Barroso-Batista J, Sousa A, Lourenço M, Bergman ML, Sobral D, Demengeot J, Xavier KB, Gordo I (2014). The first steps of adaptation of *Escherichia coli* to the gut are dominated by soft sweeps. PLoS Genet.

