# Supplementary material for: Genome analysis of E. coli isolated from Crohn’s disease patients
Source: BMC Genomics. 2017 Jul 19;18:544. doi: 10.1186/s12864-017-3917-x (PMC5517970; doi:10.1186/s12864-017-3917-x)
Supplement: Supplementary file 4 — (A) Mauve alignment of CD-E. coli isolates from one patient (alignment made for RCE01, RCE02, RCE03, RCE03 and RCE06). (B) Heatmap of the phylogenetic distance between E. coli strains. Distances between strains are calculated as the median distance of core genes (see Methods). Phylogroups of E. coli are designated (A, B1, E, B2, D, F). RCE03 is not shown because of its low similarity to other E. coli strains. (DOC 4350 kb) [file 12864_2017_3917_MOESM4_ESM.doc]

**Additional file 4A. Mauve alignment of CD-*E. coli* isolates from one patient.**

Genome assemblies from one patient were aligned using Mauve 20150226 (the Darling lab, <http://darlinglab.org/mauve/mauve.html> ) by progressiveMauve tool with default parameters.

Alignment files are given on the GitHub data depository for the current paper (Mauve_align.rar).

**Alignments from RCE01 patient.**


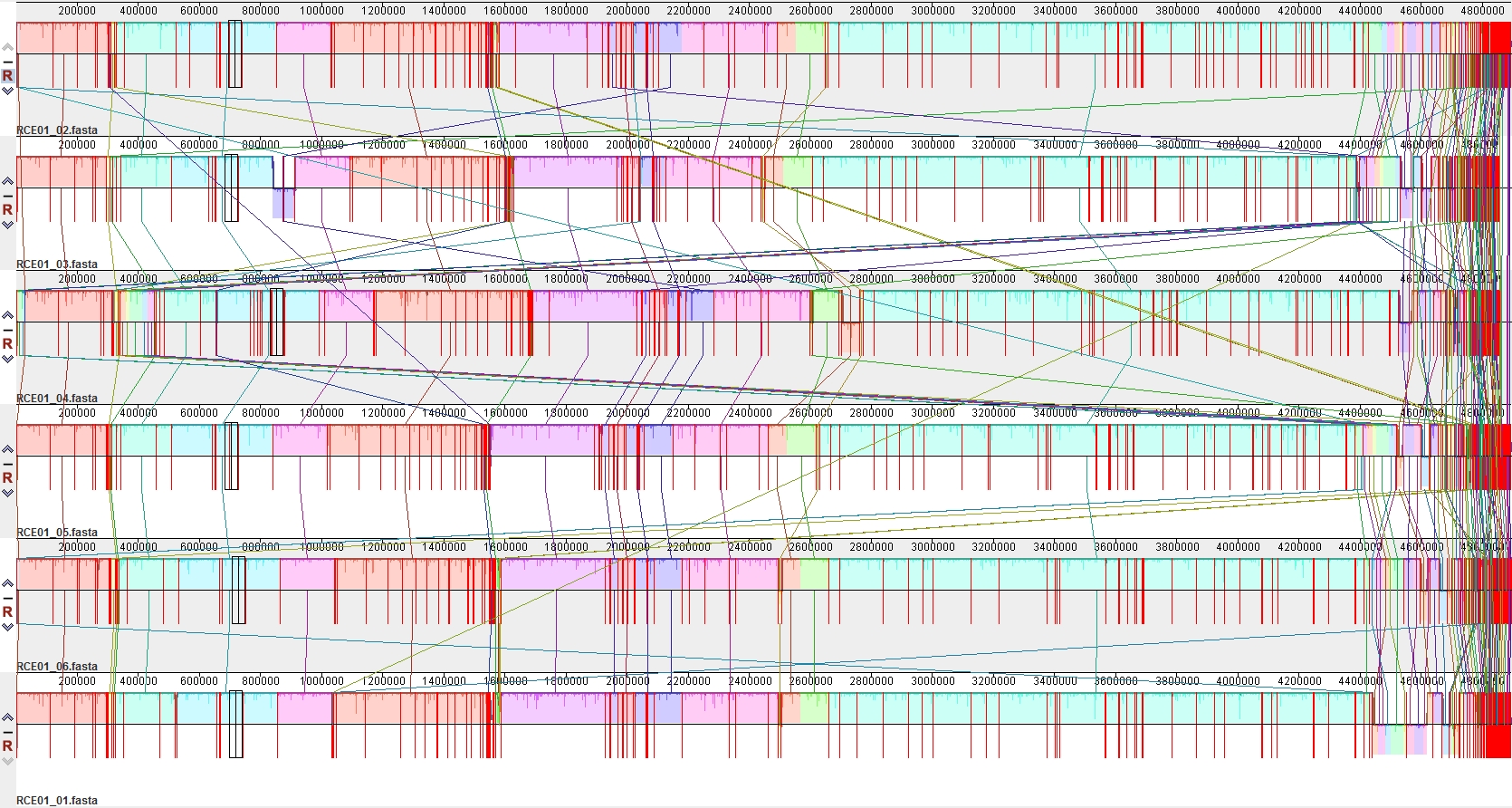


**Alignments from RCE02 patient.**


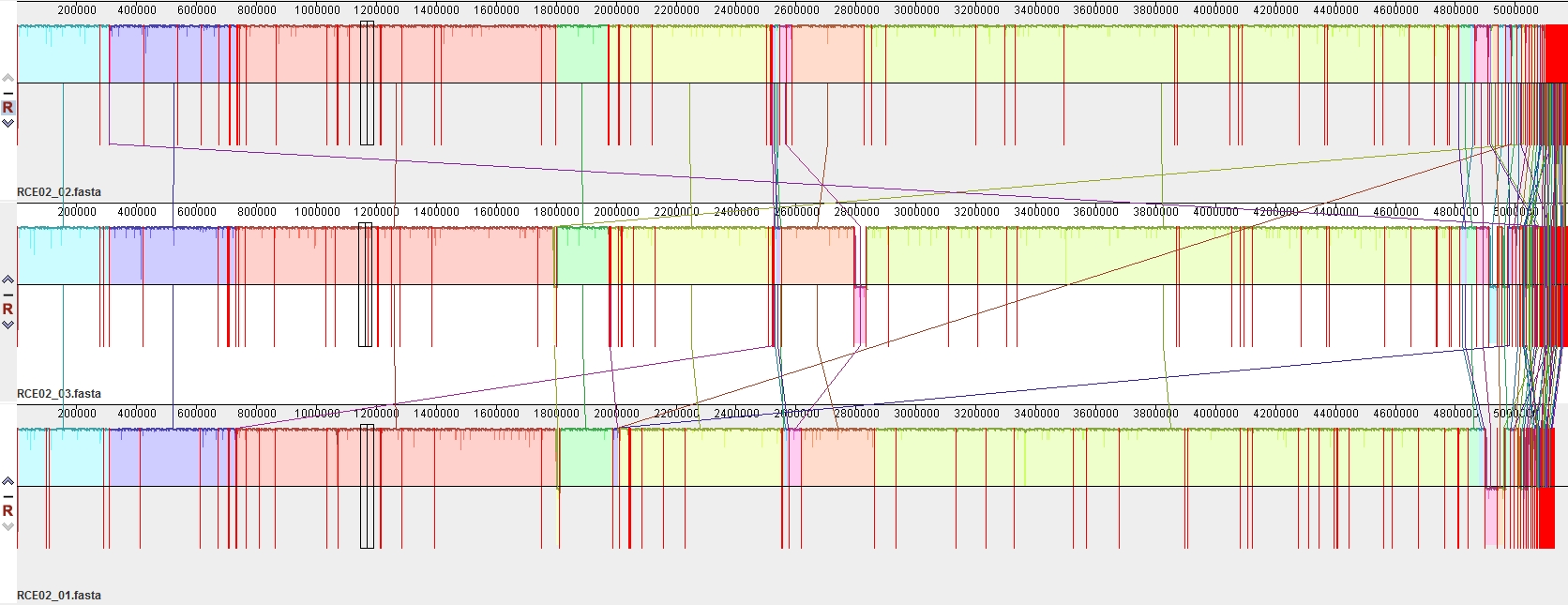


**Alignments from RCE03 patient.
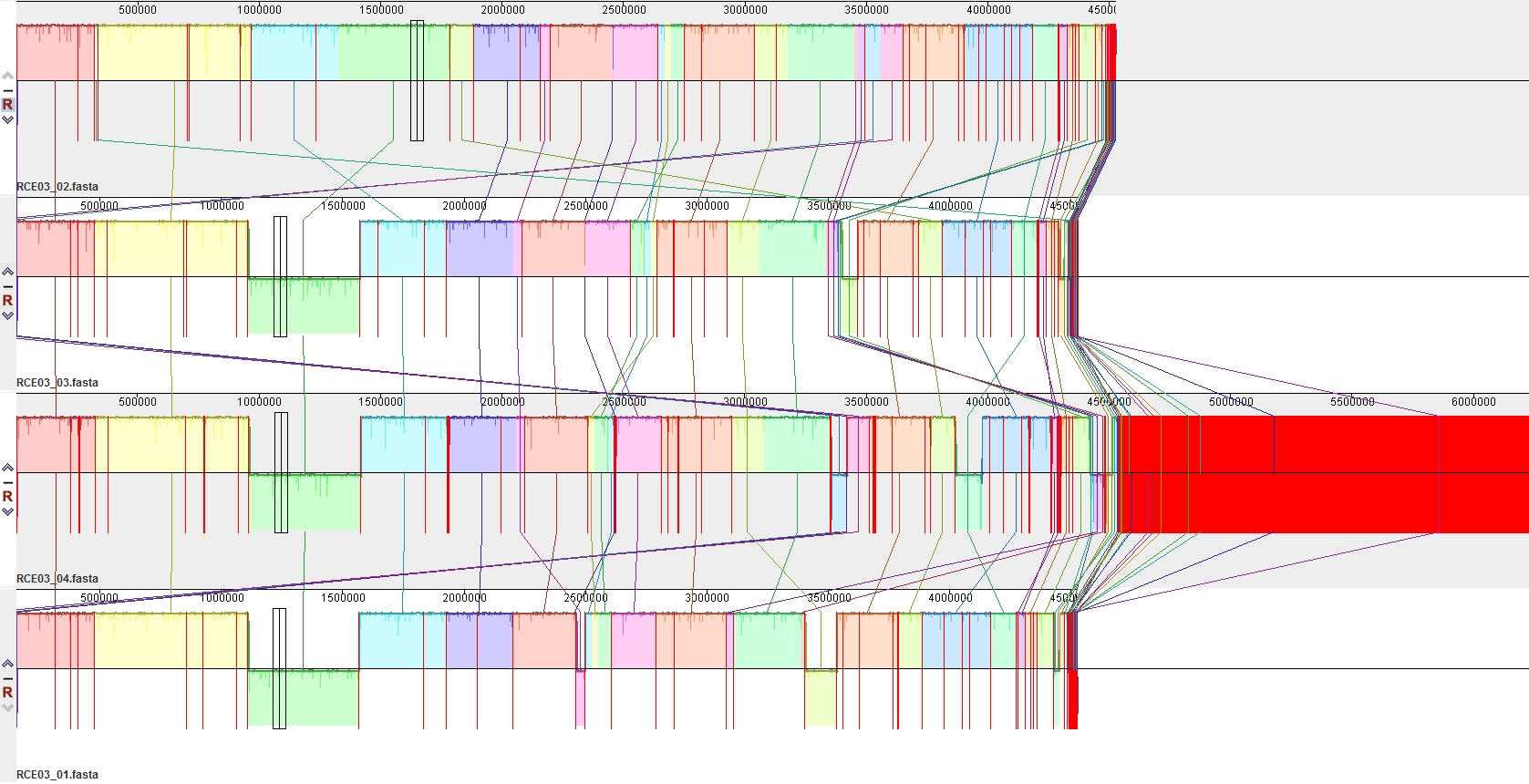
**

**Alignments from RCE04 patient.**

**
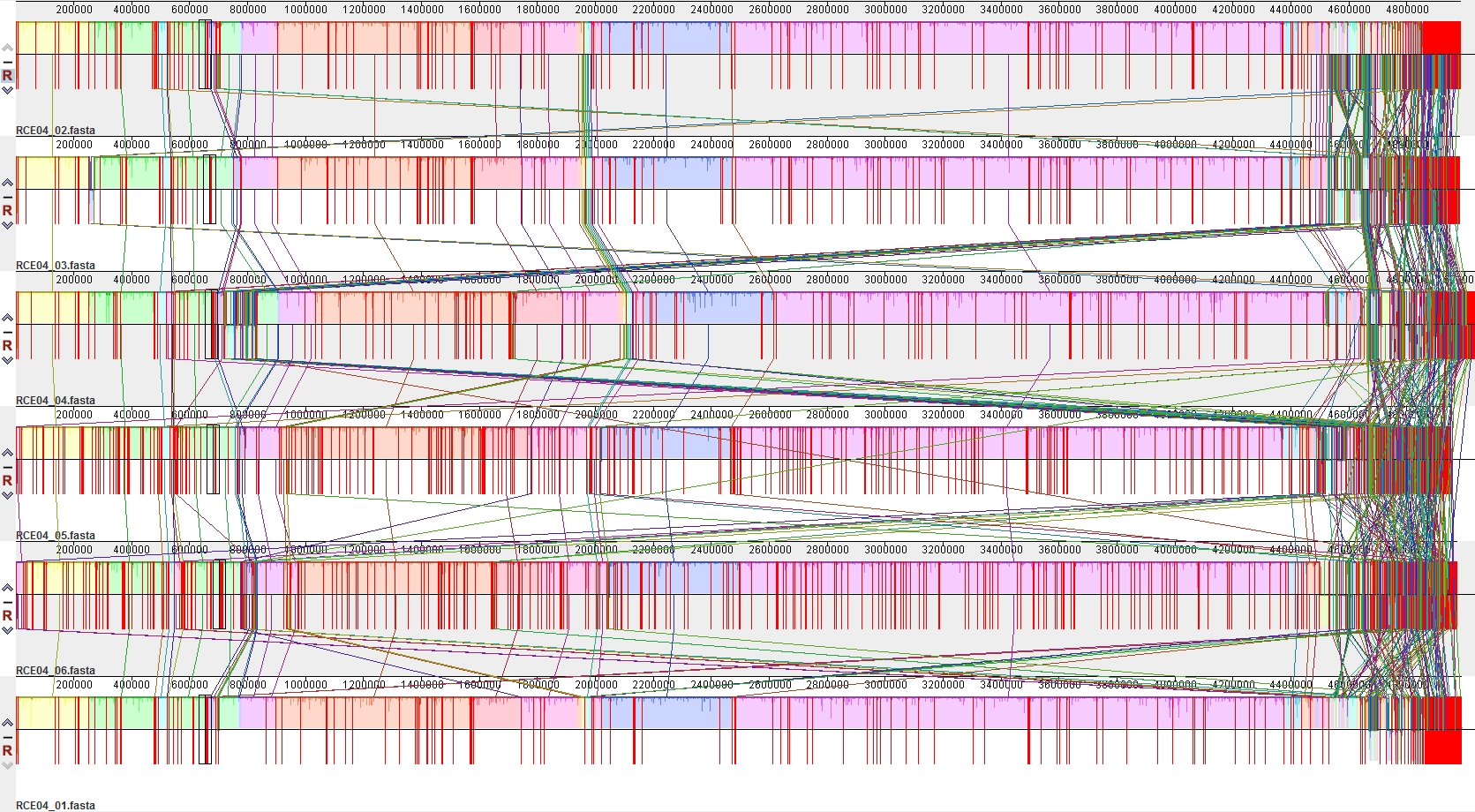
**

**Alignments from RCE06 patient.**


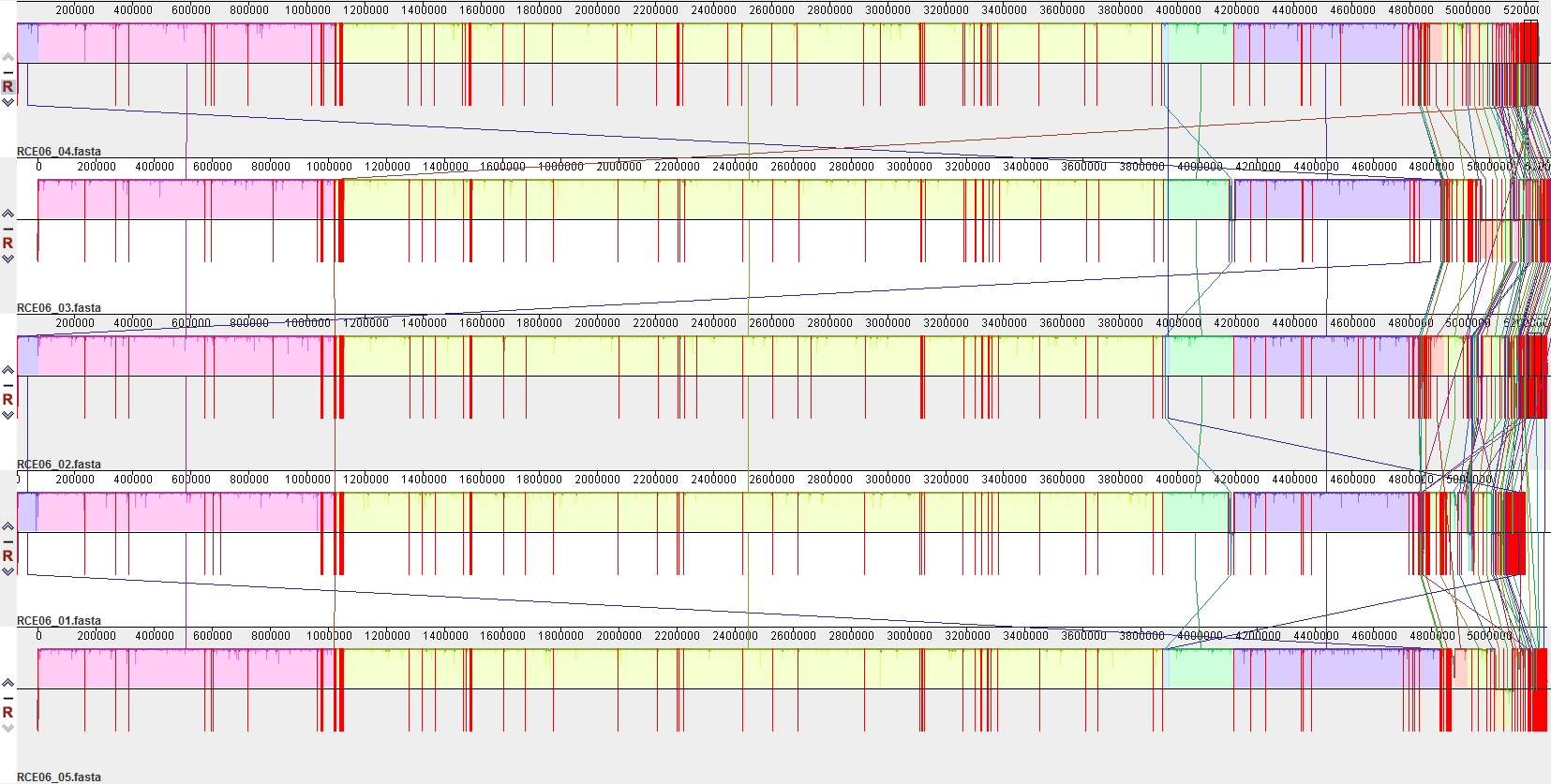


**Additional file 4.**Heatmap of phylogenetic distance between *E. coli* strains. . Distances between strains are calculated as the median distance of core genes (see Methods). Phylogroups of *E. coli* are designated (A, B1, E, B2, D, F). RCE03 isn`t shown because of great dissimilarity with other *E. coli* strains

**Color Key and Histogram**
