# Supplementary material for: Genome analysis of E. coli isolated from Crohn’s disease patients
Source: BMC Genomics. 2017 Jul 19;18:544. doi: 10.1186/s12864-017-3917-x (PMC5517970; doi:10.1186/s12864-017-3917-x)
Supplement: Supplementary file 5 — Phage sensitivity test of E. coli CD-isolates (see Methods). (DOC 45 kb) [file 12864_2017_3917_MOESM5_ESM.doc]

**Additional file 5. Phage resistance test of CD-isolated E. coli** strains (see Methods).

The phage resistance test of CD-associated strains (Supplementary Table 10) revealed that the number of P1 phage genes in the genome was 2–52 for the resistant strains and 0–75 for the sensitive strains. Strains both sensitive and resistant to the T4 phage had no genes of this phage in their genomes. All tested strains had different phage sensitivity patterns. Therefore, the presence or absence of genes of a phage in *E. coli* genome cannot be interpreted as the evidence of the strain sensitivity (resp., resistance) to this phage.

|  | Strain | dsDNA virulent  Т4 -8369 | dsDNA virulent  P1vir | temperate λcII857 | ssDNA virulent  phiX174 | | ssDNA virulent  lphi7 | | ssDNA male-specific  M13 | lphi7S1, S.typhimurium –specific (E. coli is resistant) |
| --- | --- | --- | --- | --- | --- | --- | --- | --- | --- | --- |
|  |  | spot-test | spot-test | spot-test | cross*-*streaking test | spot-test | cross*-*streaking test | spot- test | spot- test | spot- test |
|  | E.coli K-12 GA120 | R | R | R | S | S | S | S | R | R |
| 1 | RCE02-03 | R | R | R | S | S | S | S | R | R |
| 2 | RCE06-03 | R | S | R | S | S | S | S | R | R |
| 3 | RCE04-04 | S | R | R | S | S | S | S | R | R |
| 4 | RCE10 | R | R | R | S | R | S | R | R | R |
| 5 | RCE03-02 | R | S | S | S | S | S | S | R | R |
| 6 | RCE07 | S | S | R | S | S | S | S | R | R |
| 7 | RCE01-01 | R | S | R | S | R | S | R | R | R |

S – sensitive, R – resistant

Number of genes of phages used in resistance test identified in the genomes of CD- isolated E. coli strains

| strain | RCE01 | RCE02 | RCE03 | RCE04 | RCE05 | RCE06 | RCE07 | RCE08 | RCE10 | RCE11 |
| --- | --- | --- | --- | --- | --- | --- | --- | --- | --- | --- |
| PHAGE_T4 | 0 | 0 | 0 | 0 | 0 | 0 | 0 | 0 | 0 | 0 |
| PHAGE_ P1 | 75 | 52 | 2 | 12 | 89 | 1 | 0 | 0 | 2 | 37 |
| PHAGE_lambda | 8 | 0 | 1 | 2 | 1 | 0 | 2 | 1 | 1 | 1 |
