# Supplementary material for: Genome analysis of E. coli isolated from Crohn’s disease patients
Source: BMC Genomics. 2017 Jul 19;18:544. doi: 10.1186/s12864-017-3917-x (PMC5517970; doi:10.1186/s12864-017-3917-x)
Supplement: Supplementary file 7 — plLF82 homologs in CD-associated E. coli. (A) Sequence similarity of CD isolates with the plasmid pLF82 (100 kb), (B) and (C) functions of proteins common between pLF82 or pJJ1886_4 and CD isolates The functions were obtained from original annotation, complemented by Prokka annotation and BLAST protein similarity search. (D) Sequence similarity of commensal E. coli isolates with plasmids plLF82 and pJJ1886_4 (script for the data is described in supplementary data https://github.com/paraslonic/Rakitina_etal_Crohn_paper/tree/master/plasmidCoverage [41]. (DOC 228 kb) [file 12864_2017_3917_MOESM7_ESM.doc]

**Additional file 7**. plLF82 homologs within CD-associated *E. coli*. (A) Sequence similarity of CD isolates with the plasmids plLF82 and pJJ1886_4. (B) and (C) functions of proteins common between plLF82 or pJJ1886_4 and CD isolates. The functions were obtained from original annotation, complemented by Prokka annotation and BLAST protein similarity search (D) Sequence similarity of commensal *E. coli* isolates with plasmids plLF82 and pJJ1886_4 (script for the data is described in supplementary data <https://github.com/paraslonic/Rakitina_etal_Crohn_paper/tree/master/plasmidCoverage>. (E) top blastn search results for pLF82 (nr database, default search parameters) (F) blastn search results for pjj1886_4 (nr database, default search parameters)

**A**

| **Genome / assembled genome** | **Reference plasmid** | **Sequence**  **coverage, %** | **Sequence**  **homology, %** |
| --- | --- | --- | --- |
| RCE01 | plLF82 (100 kb) | 73 | 98 |
| RCE02 | pJJ1886_4 (55.9 kb) | 88 | 99 |
| RCE03 | plLF82 (100 kb) | 86 | 98 |
| RCE04 | plLF82 (100 kb) | 89 | 98 |
| CD_B2 55-1-AU4 | plLF82 (100 kb) | 89 | 98 |
| CD_B2 69-1-TI1 | pJJ1886_4 (55.9 kb) | 88 | 96 |
| CD_B2 69-1-AU1 | pJJ1886_4 (55.9 kb) | 88 | 96 |
| CD_B2 70-2-TI12 | pJJ1886_4 (55.9 kb) | 88 | 96 |

**B**

| **Number of proteins** | **Function of proteins common between plLF82 and CD genomes** |
| --- | --- |
| 32 | DNA maintenance and transfer (nucleotide base synthesis, chaperones, integrases, recombinases, conjugation etc) |
| 15 | phage proteins |
| 3 | antibiotic and multidrug-resistance |
| 7 | membrane proteins (secretion/hemagglutination, putative lipoprotein serine proteinase, alpha-hemolysin translocation ATP-binding protein, putative ABC transporter from Salmonella) |
| 2 | subunits of cobaltochelatase, B12 synthesis |
| 2 | sugar metabolism |
| 1 of each | enterotoxin, Bacterial Ig-like domain-containing protein, glutaredoxin, phlagellin transcrcription repressor and tRNA-syntase |
| 33 | hypothetical protein |

**C**

| **Number of proteins** | **Function of proteins common between pJJ1886_4 and CD genomes** |
| --- | --- |
| 24 | DNA maintenance (various functions) |
| 48 | hypothetical proteins |
| 2 | transcriptional regulator |
| 1 of each | membrane, tellurite resistance, type IV secretion protein |

D

| strain | pjj1886_4 plasmid coverage, % | pLF82 plasmid coverage, % |
| --- | --- | --- |
| TW15838 | 0.0 | 4.4 |
| C321 | 0.0 | 4.1 |
| CS01 | 0.0 | 5.1 |
| CS02 | 0.0 | 4.1 |
| CS03 | 0.0 | 4.1 |
| CS04 | 0.0 | 3.1 |
| CS05 | 0.0 | 3.1 |
| CS06 | 0.0 | 4.1 |
| ERR152024 | 0.0 | 3.1 |
| ERR152025 | 0.0 | 4.3 |
| ERR152026 | 0.0 | 3.1 |
| ERR152027 | 0.0 | 4.1 |
| ERR152028 | 0.0 | 3.4 |
| ERR152029 | 0.0 | 3.4 |
| ERR152030 | 0.0 | 3.8 |
| ERR152031 | 0.0 | 3.8 |
| ERR152032 | 0.0 | 3.8 |
| ERR152033 | 0.0 | 3.1 |
| ERR181299 | 0.1 | 4.3 |
| ERR181301 | 0.2 | 4.7 |
| ED1a_uid59379 | 1.7 | 3.3 |
| HS_uid58393 | 0.0 | 3.4 |
| IAI1_uid59377 | 0.0 | 3.8 |
| SE11_uid59425 | 0.0 | 4.6 |

E

| **Description** | [Max score](https://blast.ncbi.nlm.nih.gov/Blast.cgi?CMD=Get&ALIGNDB_BATCH_ID=413927782&ALIGNDB_CGI_HOST=blast.be-md.ncbi.nlm.nih.gov&ALIGNDB_CGI_PATH=/ALIGNDB/alndb_asn.cgi&ALIGNDB_MASTER_ALIAS=SD_ALIGNDB_MASTER&ALIGNDB_MAX_ROWS=100&ALIGNDB_ORDER_CLAUSE=seq_evalue asc,aln_id asc&ALIGNDB_WHERE_CLAUSE=seq_evalue is not null&ALIGNMENTS=100&ALIGNMENT_VIEW=Pairwise&DATABASE_SORT=0&DESCRIPTIONS=100&DYNAMIC_FORMAT=on&FIRST_QUERY_NUM=0&FORMAT_OBJECT=Alignment&FORMAT_PAGE_TARGET=&FORMAT_TYPE=HTML&GET_SEQUENCE=yes&I_THRESH=&LINE_LENGTH=60&MASK_CHAR=2&MASK_COLOR=1&NUM_OVERVIEW=100&PAGE=MegaBlast&QUERY_INDEX=0&QUERY_NUMBER=0&RESULTS_PAGE_TARGET=&RID=EX5FUK53013&SHOW_LINKOUT=yes&SHOW_OVERVIEW=yes&STEP_NUMBER=&USE_ALIGNDB=true&OLD_VIEW=false&DISPLAY_SORT=1&HSP_SORT=1) | [Total score](https://blast.ncbi.nlm.nih.gov/Blast.cgi?CMD=Get&ALIGNDB_BATCH_ID=413927782&ALIGNDB_CGI_HOST=blast.be-md.ncbi.nlm.nih.gov&ALIGNDB_CGI_PATH=/ALIGNDB/alndb_asn.cgi&ALIGNDB_MASTER_ALIAS=SD_ALIGNDB_MASTER&ALIGNDB_MAX_ROWS=100&ALIGNDB_ORDER_CLAUSE=seq_evalue asc,aln_id asc&ALIGNDB_WHERE_CLAUSE=seq_evalue is not null&ALIGNMENTS=100&ALIGNMENT_VIEW=Pairwise&DATABASE_SORT=0&DESCRIPTIONS=100&DYNAMIC_FORMAT=on&FIRST_QUERY_NUM=0&FORMAT_OBJECT=Alignment&FORMAT_PAGE_TARGET=&FORMAT_TYPE=HTML&GET_SEQUENCE=yes&I_THRESH=&LINE_LENGTH=60&MASK_CHAR=2&MASK_COLOR=1&NUM_OVERVIEW=100&PAGE=MegaBlast&QUERY_INDEX=0&QUERY_NUMBER=0&RESULTS_PAGE_TARGET=&RID=EX5FUK53013&SHOW_LINKOUT=yes&SHOW_OVERVIEW=yes&STEP_NUMBER=&USE_ALIGNDB=true&OLD_VIEW=false&DISPLAY_SORT=2&HSP_SORT=1) | [Query cover](https://blast.ncbi.nlm.nih.gov/Blast.cgi?CMD=Get&ALIGNDB_BATCH_ID=413927782&ALIGNDB_CGI_HOST=blast.be-md.ncbi.nlm.nih.gov&ALIGNDB_CGI_PATH=/ALIGNDB/alndb_asn.cgi&ALIGNDB_MASTER_ALIAS=SD_ALIGNDB_MASTER&ALIGNDB_MAX_ROWS=100&ALIGNDB_ORDER_CLAUSE=seq_evalue asc,aln_id asc&ALIGNDB_WHERE_CLAUSE=seq_evalue is not null&ALIGNMENTS=100&ALIGNMENT_VIEW=Pairwise&DATABASE_SORT=0&DESCRIPTIONS=100&DYNAMIC_FORMAT=on&FIRST_QUERY_NUM=0&FORMAT_OBJECT=Alignment&FORMAT_PAGE_TARGET=&FORMAT_TYPE=HTML&GET_SEQUENCE=yes&I_THRESH=&LINE_LENGTH=60&MASK_CHAR=2&MASK_COLOR=1&NUM_OVERVIEW=100&PAGE=MegaBlast&QUERY_INDEX=0&QUERY_NUMBER=0&RESULTS_PAGE_TARGET=&RID=EX5FUK53013&SHOW_LINKOUT=yes&SHOW_OVERVIEW=yes&STEP_NUMBER=&USE_ALIGNDB=true&OLD_VIEW=false&DISPLAY_SORT=4&HSP_SORT=0) | [E value](https://blast.ncbi.nlm.nih.gov/Blast.cgi?CMD=Get&ALIGNDB_BATCH_ID=413927782&ALIGNDB_CGI_HOST=blast.be-md.ncbi.nlm.nih.gov&ALIGNDB_CGI_PATH=/ALIGNDB/alndb_asn.cgi&ALIGNDB_MASTER_ALIAS=SD_ALIGNDB_MASTER&ALIGNDB_MAX_ROWS=100&ALIGNDB_ORDER_CLAUSE=seq_evalue asc,aln_id asc&ALIGNDB_WHERE_CLAUSE=seq_evalue is not null&ALIGNMENTS=100&ALIGNMENT_VIEW=Pairwise&DATABASE_SORT=0&DESCRIPTIONS=100&DYNAMIC_FORMAT=on&FIRST_QUERY_NUM=0&FORMAT_OBJECT=Alignment&FORMAT_PAGE_TARGET=&FORMAT_TYPE=HTML&GET_SEQUENCE=yes&I_THRESH=&LINE_LENGTH=60&MASK_CHAR=2&MASK_COLOR=1&NUM_OVERVIEW=100&PAGE=MegaBlast&QUERY_INDEX=0&QUERY_NUMBER=0&RESULTS_PAGE_TARGET=&RID=EX5FUK53013&SHOW_LINKOUT=yes&SHOW_OVERVIEW=yes&STEP_NUMBER=&USE_ALIGNDB=true&OLD_VIEW=false&DISPLAY_SORT=0&HSP_SORT=0) | [Ident](https://blast.ncbi.nlm.nih.gov/Blast.cgi?CMD=Get&ALIGNDB_BATCH_ID=413927782&ALIGNDB_CGI_HOST=blast.be-md.ncbi.nlm.nih.gov&ALIGNDB_CGI_PATH=/ALIGNDB/alndb_asn.cgi&ALIGNDB_MASTER_ALIAS=SD_ALIGNDB_MASTER&ALIGNDB_MAX_ROWS=100&ALIGNDB_ORDER_CLAUSE=seq_evalue asc,aln_id asc&ALIGNDB_WHERE_CLAUSE=seq_evalue is not null&ALIGNMENTS=100&ALIGNMENT_VIEW=Pairwise&DATABASE_SORT=0&DESCRIPTIONS=100&DYNAMIC_FORMAT=on&FIRST_QUERY_NUM=0&FORMAT_OBJECT=Alignment&FORMAT_PAGE_TARGET=&FORMAT_TYPE=HTML&GET_SEQUENCE=yes&I_THRESH=&LINE_LENGTH=60&MASK_CHAR=2&MASK_COLOR=1&NUM_OVERVIEW=100&PAGE=MegaBlast&QUERY_INDEX=0&QUERY_NUMBER=0&RESULTS_PAGE_TARGET=&RID=EX5FUK53013&SHOW_LINKOUT=yes&SHOW_OVERVIEW=yes&STEP_NUMBER=&USE_ALIGNDB=true&DISPLAY_SORT=3&HSP_SORT=3) | **Accession** |
| --- | --- | --- | --- | --- | --- | --- |
| Escherichia coli LF82 plasmid, complete sequence | 200100 | 200100 | 100% | 0 | 100% | [CU638872.1](https://www.ncbi.nlm.nih.gov/nucleotide/220979952?report=genbank&log$=nucltop&blast_rank=1&RID=EU0V6DDW013) |
| Escherichia coli strain FMU073332 plasmid pEcoFMU073332c, complete sequence | 70031 | 174400 | 90% | 0 | 99% | [CP017847.1](https://www.ncbi.nlm.nih.gov/nucleotide/1109521706?report=genbank&log$=nucltop&blast_rank=2&RID=EU0V6DDW013) |
| Escherichia coli strain AR_0104 plasmid unitig_6, complete sequence | 31536 | 169700 | 90% | 0 | 99% | [CP020118.1](https://www.ncbi.nlm.nih.gov/nucleotide/1163079699?report=genbank&log$=nucltop&blast_rank=3&RID=EU0V6DDW013) |
| Escherichia coli strain G749 plasmid pG749_2, complete sequence | 57350 | 161800 | 89% | 0 | 97% | [CP014490.1](https://www.ncbi.nlm.nih.gov/nucleotide/999949383?report=genbank&log$=nucltop&blast_rank=4&RID=EU0V6DDW013) |
| Escherichia coli H89 plasmid pECOH89, complete sequence | 34049 | 157400 | 86% | 0 | 98% | [HG530657.1](https://www.ncbi.nlm.nih.gov/nucleotide/602616593?report=genbank&log$=nucltop&blast_rank=5&RID=EU0V6DDW013) |
| Escherichia coli plasmid pV234-a DNA, contig: V234-a_scaffold_1, strain: V234 | 33870 | 158600 | 86% | 0 | 98% | [LC056430.1](https://www.ncbi.nlm.nih.gov/nucleotide/1001621979?report=genbank&log$=nucltop&blast_rank=6&RID=EU0V6DDW013) |
| [Escherichia coli strain 243 plasmid AnCo1, complete sequence](https://blast.ncbi.nlm.nih.gov/Blast.cgi?CMD=Get&ALIGNDB_BATCH_ID=413700402&ALIGNDB_CGI_HOST=blast.be-md.ncbi.nlm.nih.gov&ALIGNDB_CGI_PATH=/ALIGNDB/alndb_asn.cgi&ALIGNDB_MASTER_ALIAS=SD_ALIGNDB_MASTER&ALIGNDB_MAX_ROWS=100&ALIGNDB_ORDER_CLAUSE=seq_evalue%252#alnHdr_1154349931) | 28378 | 159000 | 86% | 0 | 98% | [KY515224.1](https://www.ncbi.nlm.nih.gov/nucleotide/1154349931?report=genbank&log$=nucltop&blast_rank=7&RID=EU0V6DDW013) |
| [Escherichia coli strain Ecol_AZ162 plasmid pECAZ162_2, complete sequence](https://blast.ncbi.nlm.nih.gov/Blast.cgi?CMD=Get&ALIGNDB_BATCH_ID=413700402&ALIGNDB_CGI_HOST=blast.be-md.ncbi.nlm.nih.gov&ALIGNDB_CGI_PATH=/ALIGNDB/alndb_asn.cgi&ALIGNDB_MASTER_ALIAS=SD_ALIGNDB_MASTER&ALIGNDB_MAX_ROWS=100&ALIGNDB_ORDER_CLAUSE=seq_evalue%252#alnHdr_1151225591) | 33959 | 157500 | 85% | 0 | 98% | [CP019013.1](https://www.ncbi.nlm.nih.gov/nucleotide/1151225591?report=genbank&log$=nucltop&blast_rank=8&RID=EU0V6DDW013) |
| [Escherichia coli strain 210205630 plasmid pSLy3, complete sequence](https://blast.ncbi.nlm.nih.gov/Blast.cgi?CMD=Get&ALIGNDB_BATCH_ID=413700402&ALIGNDB_CGI_HOST=blast.be-md.ncbi.nlm.nih.gov&ALIGNDB_CGI_PATH=/ALIGNDB/alndb_asn.cgi&ALIGNDB_MASTER_ALIAS=SD_ALIGNDB_MASTER&ALIGNDB_MAX_ROWS=100&ALIGNDB_ORDER_CLAUSE=seq_evalue%252#alnHdr_1042332339) | 51402 | 151300 | 84% | 0 | 97% | [CP015915.1](https://www.ncbi.nlm.nih.gov/nucleotide/1042332339?report=genbank&log$=nucltop&blast_rank=9&RID=EU0V6DDW013) |
| [Escherichia coli strain 244 plasmid AnCo2, complete sequence](https://blast.ncbi.nlm.nih.gov/Blast.cgi?CMD=Get&ALIGNDB_BATCH_ID=413700402&ALIGNDB_CGI_HOST=blast.be-md.ncbi.nlm.nih.gov&ALIGNDB_CGI_PATH=/ALIGNDB/alndb_asn.cgi&ALIGNDB_MASTER_ALIAS=SD_ALIGNDB_MASTER&ALIGNDB_MAX_ROWS=100&ALIGNDB_ORDER_CLAUSE=seq_evalue%252#alnHdr_1154350066) | 33863 | 155700 | 84% | 0 | 98% | [KY515225.1](https://www.ncbi.nlm.nih.gov/nucleotide/1154350066?report=genbank&log$=nucltop&blast_rank=10&RID=EU0V6DDW013) |
| [Escherichia coli plasmid pMRY16-002_2 DNA, complete genome, strain: 20Ec-P-124](https://blast.ncbi.nlm.nih.gov/Blast.cgi?CMD=Get&ALIGNDB_BATCH_ID=413700402&ALIGNDB_CGI_HOST=blast.be-md.ncbi.nlm.nih.gov&ALIGNDB_CGI_PATH=/ALIGNDB/alndb_asn.cgi&ALIGNDB_MASTER_ALIAS=SD_ALIGNDB_MASTER&ALIGNDB_MAX_ROWS=100&ALIGNDB_ORDER_CLAUSE=seq_evalue%252#alnHdr_1174074975) | 33246 | 150500 | 84% | 0 | 97% | [AP017612.1](https://www.ncbi.nlm.nih.gov/nucleotide/1174074975?report=genbank&log$=nucltop&blast_rank=11&RID=EU0V6DDW013) |
| [Escherichia coli strain D4 plasmid A, complete genome](https://blast.ncbi.nlm.nih.gov/Blast.cgi?CMD=Get&ALIGNDB_BATCH_ID=413700402&ALIGNDB_CGI_HOST=blast.be-md.ncbi.nlm.nih.gov&ALIGNDB_CGI_PATH=/ALIGNDB/alndb_asn.cgi&ALIGNDB_MASTER_ALIAS=SD_ALIGNDB_MASTER&ALIGNDB_MAX_ROWS=100&ALIGNDB_ORDER_CLAUSE=seq_evalue%252#alnHdr_1114150232) | 51790 | 152900 | 83% | 0 | 98% | [CP010144.1](https://www.ncbi.nlm.nih.gov/nucleotide/1114150232?report=genbank&log$=nucltop&blast_rank=12&RID=EU0V6DDW013) |
| [Escherichia coli strain D9 plasmid A, complete genome](https://blast.ncbi.nlm.nih.gov/Blast.cgi?CMD=Get&ALIGNDB_BATCH_ID=413700402&ALIGNDB_CGI_HOST=blast.be-md.ncbi.nlm.nih.gov&ALIGNDB_CGI_PATH=/ALIGNDB/alndb_asn.cgi&ALIGNDB_MASTER_ALIAS=SD_ALIGNDB_MASTER&ALIGNDB_MAX_ROWS=100&ALIGNDB_ORDER_CLAUSE=seq_evalue%252#alnHdr_1114173486) | 51646 | 152900 | 83% | 0 | 98% | [CP010153.1](https://www.ncbi.nlm.nih.gov/nucleotide/1114173486?report=genbank&log$=nucltop&blast_rank=13&RID=EU0V6DDW013) |
| [Escherichia coli O104:H4 str. 2009EL-2050 plasmid p09EL50, complete sequence](https://blast.ncbi.nlm.nih.gov/Blast.cgi?CMD=Get&ALIGNDB_BATCH_ID=413700402&ALIGNDB_CGI_HOST=blast.be-md.ncbi.nlm.nih.gov&ALIGNDB_CGI_PATH=/ALIGNDB/alndb_asn.cgi&ALIGNDB_MASTER_ALIAS=SD_ALIGNDB_MASTER&ALIGNDB_MAX_ROWS=100&ALIGNDB_ORDER_CLAUSE=seq_evalue%252#alnHdr_406780272) | 40472 | 144600 | 83% | 0 | 97% | [CP003298.1](https://www.ncbi.nlm.nih.gov/nucleotide/406780272?report=genbank&log$=nucltop&blast_rank=14&RID=EU0V6DDW013) |
| [Escherichia coli strain Ecol_881 plasmid pEC881_2, complete sequence](https://blast.ncbi.nlm.nih.gov/Blast.cgi?CMD=Get&ALIGNDB_BATCH_ID=413700402&ALIGNDB_CGI_HOST=blast.be-md.ncbi.nlm.nih.gov&ALIGNDB_CGI_PATH=/ALIGNDB/alndb_asn.cgi&ALIGNDB_MASTER_ALIAS=SD_ALIGNDB_MASTER&ALIGNDB_MAX_ROWS=100&ALIGNDB_ORDER_CLAUSE=seq_evalue%252#alnHdr_1151181235) | 33639 | 150900 | 83% | 0 | 98% | [CP019027.1](https://www.ncbi.nlm.nih.gov/nucleotide/1151181235?report=genbank&log$=nucltop&blast_rank=15&RID=EU0V6DDW013) |
| [Escherichia coli strain MG1655 YFP plasmid ESBL242, complete sequence](https://blast.ncbi.nlm.nih.gov/Blast.cgi?CMD=Get&ALIGNDB_BATCH_ID=413700402&ALIGNDB_CGI_HOST=blast.be-md.ncbi.nlm.nih.gov&ALIGNDB_CGI_PATH=/ALIGNDB/alndb_asn.cgi&ALIGNDB_MASTER_ALIAS=SD_ALIGNDB_MASTER&ALIGNDB_MAX_ROWS=100&ALIGNDB_ORDER_CLAUSE=seq_evalue%252#alnHdr_848623755) | 30969 | 151200 | 80% | 0 | 98% | [KP792123.1](https://www.ncbi.nlm.nih.gov/nucleotide/848623755?report=genbank&log$=nucltop&blast_rank=16&RID=EU0V6DDW013) |
| [Escherichia coli strain Ecol_AZ155 plasmid pECAZ155_2, complete sequence](https://blast.ncbi.nlm.nih.gov/Blast.cgi?CMD=Get&ALIGNDB_BATCH_ID=413700402&ALIGNDB_CGI_HOST=blast.be-md.ncbi.nlm.nih.gov&ALIGNDB_CGI_PATH=/ALIGNDB/alndb_asn.cgi&ALIGNDB_MASTER_ALIAS=SD_ALIGNDB_MASTER&ALIGNDB_MAX_ROWS=100&ALIGNDB_ORDER_CLAUSE=seq_evalue%252#alnHdr_1151203282) | 33778 | 132900 | 75% | 0 | 98% | [CP019002.1](https://www.ncbi.nlm.nih.gov/nucleotide/1151203282?report=genbank&log$=nucltop&blast_rank=17&RID=EU0V6DDW013) |
| [Enterobacter cloacae strain CY01 plasmid pCY-CTX, complete sequence](https://blast.ncbi.nlm.nih.gov/Blast.cgi?CMD=Get&ALIGNDB_BATCH_ID=413700402&ALIGNDB_CGI_HOST=blast.be-md.ncbi.nlm.nih.gov&ALIGNDB_CGI_PATH=/ALIGNDB/alndb_asn.cgi&ALIGNDB_MASTER_ALIAS=SD_ALIGNDB_MASTER&ALIGNDB_MAX_ROWS=100&ALIGNDB_ORDER_CLAUSE=seq_evalue%252#alnHdr_1025730442) | 11912 | 51019 | 65% | 0 | 82% | [KX015668.1](https://www.ncbi.nlm.nih.gov/nucleotide/1025730442?report=genbank&log$=nucltop&blast_rank=18&RID=EU0V6DDW013) |
| [Escherichia coli strain S56 plasmid B, complete sequence](https://blast.ncbi.nlm.nih.gov/Blast.cgi?CMD=Get&ALIGNDB_BATCH_ID=413700402&ALIGNDB_CGI_HOST=blast.be-md.ncbi.nlm.nih.gov&ALIGNDB_CGI_PATH=/ALIGNDB/alndb_asn.cgi&ALIGNDB_MASTER_ALIAS=SD_ALIGNDB_MASTER&ALIGNDB_MAX_ROWS=100&ALIGNDB_ORDER_CLAUSE=seq_evalue%252#alnHdr_1114345345) | 16096 | 62277 | 63% | 0 | 88% | [CP010244.1](https://www.ncbi.nlm.nih.gov/nucleotide/1114345345?report=genbank&log$=nucltop&blast_rank=19&RID=EU0V6DDW013) |
| Salmonella phage SSU5, complete genome | 10368 | 51151 | 63% | 0 | 82% | [JQ965645.1](https://www.ncbi.nlm.nih.gov/nucleotide/390013835?report=genbank&log$=nucltop&blast_rank=20&RID=EU0V6DDW013) |
| [Salmonella enterica subsp. enterica serovar Stanleyville str. CFSAN000624 strain SARB61 plasmid pSARB26_01, complete sequence](https://blast.ncbi.nlm.nih.gov/Blast.cgi?CMD=Get&ALIGNDB_BATCH_ID=413700402&ALIGNDB_CGI_HOST=blast.be-md.ncbi.nlm.nih.gov&ALIGNDB_CGI_PATH=/ALIGNDB/alndb_asn.cgi&ALIGNDB_MASTER_ALIAS=SD_ALIGNDB_MASTER&ALIGNDB_MAX_ROWS=100&ALIGNDB_ORDER_CLAUSE=seq_evalue%252#alnHdr_1092935905) | 8938 | 50116 | 63% | 0 | 82% | [CP017724.1](https://www.ncbi.nlm.nih.gov/nucleotide/1092935905?report=genbank&log$=nucltop&blast_rank=21&RID=EU0V6DDW013) |
| [Salmonella enterica subsp. enterica serovar Typhi strain Ty004 plasmid pTy004_01, complete sequence](https://blast.ncbi.nlm.nih.gov/Blast.cgi?CMD=Get&ALIGNDB_BATCH_ID=413700402&ALIGNDB_CGI_HOST=blast.be-md.ncbi.nlm.nih.gov&ALIGNDB_CGI_PATH=/ALIGNDB/alndb_asn.cgi&ALIGNDB_MASTER_ALIAS=SD_ALIGNDB_MASTER&ALIGNDB_MAX_ROWS=100&ALIGNDB_ORDER_CLAUSE=seq_evalue%252#alnHdr_1112882878) | 11804 | 50139 | 62% | 0 | 82% | [KX833209.1](https://www.ncbi.nlm.nih.gov/nucleotide/1112882878?report=genbank&log$=nucltop&blast_rank=22&RID=EU0V6DDW013) |
| [Citrobacter freundii strain CAV1741 plasmid pCAV1741-110, complete sequence](https://blast.ncbi.nlm.nih.gov/Blast.cgi?CMD=Get&ALIGNDB_BATCH_ID=413700402&ALIGNDB_CGI_HOST=blast.be-md.ncbi.nlm.nih.gov&ALIGNDB_CGI_PATH=/ALIGNDB/alndb_asn.cgi&ALIGNDB_MASTER_ALIAS=SD_ALIGNDB_MASTER&ALIGNDB_MAX_ROWS=100&ALIGNDB_ORDER_CLAUSE=seq_evalue%252#alnHdr_828982776) | 11727 | 49612 | 62% | 0 | 82% | [CP011655.1](https://www.ncbi.nlm.nih.gov/nucleotide/828982776?report=genbank&log$=nucltop&blast_rank=23&RID=EU0V6DDW013) |
| [Salmonella enterica subsp. enterica serovar Typhi str. CT18 plasmid pHCM2](https://blast.ncbi.nlm.nih.gov/Blast.cgi?CMD=Get&ALIGNDB_BATCH_ID=413700402&ALIGNDB_CGI_HOST=blast.be-md.ncbi.nlm.nih.gov&ALIGNDB_CGI_PATH=/ALIGNDB/alndb_asn.cgi&ALIGNDB_MASTER_ALIAS=SD_ALIGNDB_MASTER&ALIGNDB_MAX_ROWS=100&ALIGNDB_ORDER_CLAUSE=seq_evalue%252#alnHdr_16505981) | 11679 | 49792 | 62% | 0 | 82% | [AL513384.1](https://www.ncbi.nlm.nih.gov/nucleotide/16505981?report=genbank&log$=nucltop&blast_rank=24&RID=EU0V6DDW013) |
| [Enterobacter cloacae strain G6809 plasmid pG6809-1, complete sequence](https://blast.ncbi.nlm.nih.gov/Blast.cgi?CMD=Get&ALIGNDB_BATCH_ID=413700402&ALIGNDB_CGI_HOST=blast.be-md.ncbi.nlm.nih.gov&ALIGNDB_CGI_PATH=/ALIGNDB/alndb_asn.cgi&ALIGNDB_MASTER_ALIAS=SD_ALIGNDB_MASTER&ALIGNDB_MAX_ROWS=100&ALIGNDB_ORDER_CLAUSE=seq_evalue%252#alnHdr_941350113) | 11594 | 47031 | 61% | 0 | 82% | [KT345945.1](https://www.ncbi.nlm.nih.gov/nucleotide/941350113?report=genbank&log$=nucltop&blast_rank=25&RID=EU0V6DDW013) |
| [Enterobacter hormaechei subsp. oharae strain 34399 plasmid p34399-121.660kb, complete sequence](https://blast.ncbi.nlm.nih.gov/Blast.cgi?CMD=Get&ALIGNDB_BATCH_ID=413700402&ALIGNDB_CGI_HOST=blast.be-md.ncbi.nlm.nih.gov&ALIGNDB_CGI_PATH=/ALIGNDB/alndb_asn.cgi&ALIGNDB_MASTER_ALIAS=SD_ALIGNDB_MASTER&ALIGNDB_MAX_ROWS=100&ALIGNDB_ORDER_CLAUSE=seq_evalue%252#alnHdr_742993713) | 11502 | 48804 | 61% | 0 | 82% | [CP010386.1](https://www.ncbi.nlm.nih.gov/nucleotide/742993713?report=genbank&log$=nucltop&blast_rank=26&RID=EU0V6DDW013) |
| [Salmonella enterica subsp. enterica serovar Tennessee strain CFSAN001387 plasmid, complete sequence](https://blast.ncbi.nlm.nih.gov/Blast.cgi?CMD=Get&ALIGNDB_BATCH_ID=413700402&ALIGNDB_CGI_HOST=blast.be-md.ncbi.nlm.nih.gov&ALIGNDB_CGI_PATH=/ALIGNDB/alndb_asn.cgi&ALIGNDB_MASTER_ALIAS=SD_ALIGNDB_MASTER&ALIGNDB_MAX_ROWS=100&ALIGNDB_ORDER_CLAUSE=seq_evalue%252#alnHdr_1015694605) | 6111 | 48403 | 60% | 0 | 83% | [CP014995.1](https://www.ncbi.nlm.nih.gov/nucleotide/1015694605?report=genbank&log$=nucltop&blast_rank=27&RID=EU0V6DDW013) |
| [Yersinia pestis 3067 plasmid pMT1, complete sequence](https://blast.ncbi.nlm.nih.gov/Blast.cgi?CMD=Get&ALIGNDB_BATCH_ID=413700402&ALIGNDB_CGI_HOST=blast.be-md.ncbi.nlm.nih.gov&ALIGNDB_CGI_PATH=/ALIGNDB/alndb_asn.cgi&ALIGNDB_MASTER_ALIAS=SD_ALIGNDB_MASTER&ALIGNDB_MAX_ROWS=100&ALIGNDB_ORDER_CLAUSE=seq_evalue%252#alnHdr_908257872) | 6842 | 40093 | 52% | 0 | 83% | [CP006752.1](https://www.ncbi.nlm.nih.gov/nucleotide/908257872?report=genbank&log$=nucltop&blast_rank=28&RID=EU0V6DDW013) |
| [Yersinia pestis 8787 plasmid pMT1, complete sequence](https://blast.ncbi.nlm.nih.gov/Blast.cgi?CMD=Get&ALIGNDB_BATCH_ID=413700402&ALIGNDB_CGI_HOST=blast.be-md.ncbi.nlm.nih.gov&ALIGNDB_CGI_PATH=/ALIGNDB/alndb_asn.cgi&ALIGNDB_MASTER_ALIAS=SD_ALIGNDB_MASTER&ALIGNDB_MAX_ROWS=100&ALIGNDB_ORDER_CLAUSE=seq_evalue%252#alnHdr_908249461) | 6842 | 40097 | 52% | 0 | 83% | [CP006746.1](https://www.ncbi.nlm.nih.gov/nucleotide/908249461?report=genbank&log$=nucltop&blast_rank=29&RID=EU0V6DDW013) |
| [Yersinia pestis 1413 plasmid pMT, complete sequence](https://blast.ncbi.nlm.nih.gov/Blast.cgi?CMD=Get&ALIGNDB_BATCH_ID=413700402&ALIGNDB_CGI_HOST=blast.be-md.ncbi.nlm.nih.gov&ALIGNDB_CGI_PATH=/ALIGNDB/alndb_asn.cgi&ALIGNDB_MASTER_ALIAS=SD_ALIGNDB_MASTER&ALIGNDB_MAX_ROWS=100&ALIGNDB_ORDER_CLAUSE=seq_evalue%252#alnHdr_908236742) | 6842 | 40097 | 52% | 0 | 83% | [CP006760.1](https://www.ncbi.nlm.nih.gov/nucleotide/908236742?report=genbank&log$=nucltop&blast_rank=30&RID=EU0V6DDW013) |
| [Yersinia pestis 1412 plasmid pMT, complete sequence](https://blast.ncbi.nlm.nih.gov/Blast.cgi?CMD=Get&ALIGNDB_BATCH_ID=413700402&ALIGNDB_CGI_HOST=blast.be-md.ncbi.nlm.nih.gov&ALIGNDB_CGI_PATH=/ALIGNDB/alndb_asn.cgi&ALIGNDB_MASTER_ALIAS=SD_ALIGNDB_MASTER&ALIGNDB_MAX_ROWS=100&ALIGNDB_ORDER_CLAUSE=seq_evalue%252#alnHdr_908217494) | 6842 | 40097 | 52% | 0 | 83% | [CP006779.1](https://www.ncbi.nlm.nih.gov/nucleotide/908217494?report=genbank&log$=nucltop&blast_rank=31&RID=EU0V6DDW013) |
| [Yersinia pestis Pestoides G plasmid pMT sequence](https://blast.ncbi.nlm.nih.gov/Blast.cgi?CMD=Get&ALIGNDB_BATCH_ID=413700402&ALIGNDB_CGI_HOST=blast.be-md.ncbi.nlm.nih.gov&ALIGNDB_CGI_PATH=/ALIGNDB/alndb_asn.cgi&ALIGNDB_MASTER_ALIAS=SD_ALIGNDB_MASTER&ALIGNDB_MAX_ROWS=100&ALIGNDB_ORDER_CLAUSE=seq_evalue%252#alnHdr_755462046) | 6842 | 40097 | 52% | 0 | 83% | [CP010248.1](https://www.ncbi.nlm.nih.gov/nucleotide/755462046?report=genbank&log$=nucltop&blast_rank=32&RID=EU0V6DDW013) |
| [Yersinia pestis Pestoides F plasmid pMT, complete sequence](https://blast.ncbi.nlm.nih.gov/Blast.cgi?CMD=Get&ALIGNDB_BATCH_ID=413700402&ALIGNDB_CGI_HOST=blast.be-md.ncbi.nlm.nih.gov&ALIGNDB_CGI_PATH=/ALIGNDB/alndb_asn.cgi&ALIGNDB_MASTER_ALIAS=SD_ALIGNDB_MASTER&ALIGNDB_MAX_ROWS=100&ALIGNDB_ORDER_CLAUSE=seq_evalue%252#alnHdr_755363455) | 6842 | 40129 | 52% | 0 | 83% | [CP009714.1](https://www.ncbi.nlm.nih.gov/nucleotide/755363455?report=genbank&log$=nucltop&blast_rank=33&RID=EU0V6DDW013) |
| [Yersinia pestis Pestoides F plasmid MT, complete sequence](https://blast.ncbi.nlm.nih.gov/Blast.cgi?CMD=Get&ALIGNDB_BATCH_ID=413700402&ALIGNDB_CGI_HOST=blast.be-md.ncbi.nlm.nih.gov&ALIGNDB_CGI_PATH=/ALIGNDB/alndb_asn.cgi&ALIGNDB_MASTER_ALIAS=SD_ALIGNDB_MASTER&ALIGNDB_MAX_ROWS=100&ALIGNDB_ORDER_CLAUSE=seq_evalue%252#alnHdr_145212960) | 6842 | 40112 | 52% | 0 | 83% | [CP000670.1](https://www.ncbi.nlm.nih.gov/nucleotide/145212960?report=genbank&log$=nucltop&blast_rank=34&RID=EU0V6DDW013) |
| [Yersinia pestis 1522 plasmid pMT, complete sequence](https://blast.ncbi.nlm.nih.gov/Blast.cgi?CMD=Get&ALIGNDB_BATCH_ID=413700402&ALIGNDB_CGI_HOST=blast.be-md.ncbi.nlm.nih.gov&ALIGNDB_CGI_PATH=/ALIGNDB/alndb_asn.cgi&ALIGNDB_MASTER_ALIAS=SD_ALIGNDB_MASTER&ALIGNDB_MAX_ROWS=100&ALIGNDB_ORDER_CLAUSE=seq_evalue%252#alnHdr_908240976) | 6837 | 40110 | 52% | 0 | 83% | [CP006756.1](https://www.ncbi.nlm.nih.gov/nucleotide/908240976?report=genbank&log$=nucltop&blast_rank=35&RID=EU0V6DDW013) |

F

| **Description** | [Max score](https://blast.ncbi.nlm.nih.gov/Blast.cgi?CMD=Get&ALIGNDB_BATCH_ID=413927782&ALIGNDB_CGI_HOST=blast.be-md.ncbi.nlm.nih.gov&ALIGNDB_CGI_PATH=/ALIGNDB/alndb_asn.cgi&ALIGNDB_MASTER_ALIAS=SD_ALIGNDB_MASTER&ALIGNDB_MAX_ROWS=100&ALIGNDB_ORDER_CLAUSE=seq_evalue asc,aln_id asc&ALIGNDB_WHERE_CLAUSE=seq_evalue is not null&ALIGNMENTS=100&ALIGNMENT_VIEW=Pairwise&DATABASE_SORT=0&DESCRIPTIONS=100&DYNAMIC_FORMAT=on&FIRST_QUERY_NUM=0&FORMAT_OBJECT=Alignment&FORMAT_PAGE_TARGET=&FORMAT_TYPE=HTML&GET_SEQUENCE=yes&I_THRESH=&LINE_LENGTH=60&MASK_CHAR=2&MASK_COLOR=1&NUM_OVERVIEW=100&PAGE=MegaBlast&QUERY_INDEX=0&QUERY_NUMBER=0&RESULTS_PAGE_TARGET=&RID=EX5FUK53013&SHOW_LINKOUT=yes&SHOW_OVERVIEW=yes&STEP_NUMBER=&USE_ALIGNDB=true&OLD_VIEW=false&DISPLAY_SORT=1&HSP_SORT=1) | [Total score](https://blast.ncbi.nlm.nih.gov/Blast.cgi?CMD=Get&ALIGNDB_BATCH_ID=413927782&ALIGNDB_CGI_HOST=blast.be-md.ncbi.nlm.nih.gov&ALIGNDB_CGI_PATH=/ALIGNDB/alndb_asn.cgi&ALIGNDB_MASTER_ALIAS=SD_ALIGNDB_MASTER&ALIGNDB_MAX_ROWS=100&ALIGNDB_ORDER_CLAUSE=seq_evalue asc,aln_id asc&ALIGNDB_WHERE_CLAUSE=seq_evalue is not null&ALIGNMENTS=100&ALIGNMENT_VIEW=Pairwise&DATABASE_SORT=0&DESCRIPTIONS=100&DYNAMIC_FORMAT=on&FIRST_QUERY_NUM=0&FORMAT_OBJECT=Alignment&FORMAT_PAGE_TARGET=&FORMAT_TYPE=HTML&GET_SEQUENCE=yes&I_THRESH=&LINE_LENGTH=60&MASK_CHAR=2&MASK_COLOR=1&NUM_OVERVIEW=100&PAGE=MegaBlast&QUERY_INDEX=0&QUERY_NUMBER=0&RESULTS_PAGE_TARGET=&RID=EX5FUK53013&SHOW_LINKOUT=yes&SHOW_OVERVIEW=yes&STEP_NUMBER=&USE_ALIGNDB=true&OLD_VIEW=false&DISPLAY_SORT=2&HSP_SORT=1) | [Query cover](https://blast.ncbi.nlm.nih.gov/Blast.cgi?CMD=Get&ALIGNDB_BATCH_ID=413927782&ALIGNDB_CGI_HOST=blast.be-md.ncbi.nlm.nih.gov&ALIGNDB_CGI_PATH=/ALIGNDB/alndb_asn.cgi&ALIGNDB_MASTER_ALIAS=SD_ALIGNDB_MASTER&ALIGNDB_MAX_ROWS=100&ALIGNDB_ORDER_CLAUSE=seq_evalue asc,aln_id asc&ALIGNDB_WHERE_CLAUSE=seq_evalue is not null&ALIGNMENTS=100&ALIGNMENT_VIEW=Pairwise&DATABASE_SORT=0&DESCRIPTIONS=100&DYNAMIC_FORMAT=on&FIRST_QUERY_NUM=0&FORMAT_OBJECT=Alignment&FORMAT_PAGE_TARGET=&FORMAT_TYPE=HTML&GET_SEQUENCE=yes&I_THRESH=&LINE_LENGTH=60&MASK_CHAR=2&MASK_COLOR=1&NUM_OVERVIEW=100&PAGE=MegaBlast&QUERY_INDEX=0&QUERY_NUMBER=0&RESULTS_PAGE_TARGET=&RID=EX5FUK53013&SHOW_LINKOUT=yes&SHOW_OVERVIEW=yes&STEP_NUMBER=&USE_ALIGNDB=true&OLD_VIEW=false&DISPLAY_SORT=4&HSP_SORT=0) | [E value](https://blast.ncbi.nlm.nih.gov/Blast.cgi?CMD=Get&ALIGNDB_BATCH_ID=413927782&ALIGNDB_CGI_HOST=blast.be-md.ncbi.nlm.nih.gov&ALIGNDB_CGI_PATH=/ALIGNDB/alndb_asn.cgi&ALIGNDB_MASTER_ALIAS=SD_ALIGNDB_MASTER&ALIGNDB_MAX_ROWS=100&ALIGNDB_ORDER_CLAUSE=seq_evalue asc,aln_id asc&ALIGNDB_WHERE_CLAUSE=seq_evalue is not null&ALIGNMENTS=100&ALIGNMENT_VIEW=Pairwise&DATABASE_SORT=0&DESCRIPTIONS=100&DYNAMIC_FORMAT=on&FIRST_QUERY_NUM=0&FORMAT_OBJECT=Alignment&FORMAT_PAGE_TARGET=&FORMAT_TYPE=HTML&GET_SEQUENCE=yes&I_THRESH=&LINE_LENGTH=60&MASK_CHAR=2&MASK_COLOR=1&NUM_OVERVIEW=100&PAGE=MegaBlast&QUERY_INDEX=0&QUERY_NUMBER=0&RESULTS_PAGE_TARGET=&RID=EX5FUK53013&SHOW_LINKOUT=yes&SHOW_OVERVIEW=yes&STEP_NUMBER=&USE_ALIGNDB=true&OLD_VIEW=false&DISPLAY_SORT=0&HSP_SORT=0) | [Ident](https://blast.ncbi.nlm.nih.gov/Blast.cgi?CMD=Get&ALIGNDB_BATCH_ID=413927782&ALIGNDB_CGI_HOST=blast.be-md.ncbi.nlm.nih.gov&ALIGNDB_CGI_PATH=/ALIGNDB/alndb_asn.cgi&ALIGNDB_MASTER_ALIAS=SD_ALIGNDB_MASTER&ALIGNDB_MAX_ROWS=100&ALIGNDB_ORDER_CLAUSE=seq_evalue asc,aln_id asc&ALIGNDB_WHERE_CLAUSE=seq_evalue is not null&ALIGNMENTS=100&ALIGNMENT_VIEW=Pairwise&DATABASE_SORT=0&DESCRIPTIONS=100&DYNAMIC_FORMAT=on&FIRST_QUERY_NUM=0&FORMAT_OBJECT=Alignment&FORMAT_PAGE_TARGET=&FORMAT_TYPE=HTML&GET_SEQUENCE=yes&I_THRESH=&LINE_LENGTH=60&MASK_CHAR=2&MASK_COLOR=1&NUM_OVERVIEW=100&PAGE=MegaBlast&QUERY_INDEX=0&QUERY_NUMBER=0&RESULTS_PAGE_TARGET=&RID=EX5FUK53013&SHOW_LINKOUT=yes&SHOW_OVERVIEW=yes&STEP_NUMBER=&USE_ALIGNDB=true&DISPLAY_SORT=3&HSP_SORT=3) | **Accession** |
| --- | --- | --- | --- | --- | --- | --- |
| Escherichia coli JJ1886 plasmid pJJ1886_4, complete sequence | 1.03E+05 | 1.03E+05 | 100% | 0 | 100% | [CP006788.1](https://www.ncbi.nlm.nih.gov/nucleotide/554587771?report=genbank&log$=nucltop&blast_rank=1&RID=EX5FUK53013) |
| Escherichia coli isolate FP671 plasmid pHNFP671, complete sequence | 22506 | 68361 | 74% | 0 | 98% | [KP324830.1](https://www.ncbi.nlm.nih.gov/nucleotide/762041539?report=genbank&log$=nucltop&blast_rank=2&RID=EX5FUK53013) |
| Klebsiella pneumoniae strain WCHKP1511 plasmid pMCR_1511, complete sequence | 22500 | 68577 | 74% | 0 | 98% | [KX377410.1](https://www.ncbi.nlm.nih.gov/nucleotide/1098552673?report=genbank&log$=nucltop&blast_rank=3&RID=EX5FUK53013) |
| Salmonella enterica subsp. enterica serovar Typhimurium strain YL14P053 plasmid pMCR16_P053, complete sequence | 22437 | 68623 | 73% | 0 | 98% | [KY352406.1](https://www.ncbi.nlm.nih.gov/nucleotide/1142684289?report=genbank&log$=nucltop&blast_rank=4&RID=EX5FUK53013) |
| Escherichia coli strain HS102707 plasmid pHS102707, complete sequence | 15935 | 75136 | 82% | 0 | 97% | [KF701335.1](https://www.ncbi.nlm.nih.gov/nucleotide/583930872?report=genbank&log$=nucltop&blast_rank=5&RID=EX5FUK53013) |
| Escherichia coli strain DD81 plasmid pHNDD81-1, complete sequence | 8493 | 8493 | 8% | 0 | 98% | [JN232518.1](https://www.ncbi.nlm.nih.gov/nucleotide/359359384?report=genbank&log$=nucltop&blast_rank=6&RID=EX5FUK53013) |
| Uncultured bacterium plasmid pDS1, complete sequence | 8019 | 13989 | 40% | 0 | 78% | [KC170283.1](https://www.ncbi.nlm.nih.gov/nucleotide/469813508?report=genbank&log$=nucltop&blast_rank=7&RID=EX5FUK53013) |
| Klebsiella pneumoniae strain 565 plasmid PKPCAPSS, complete sequence | 6397 | 10215 | 10% | 0 | 98% | [KP008371.1](https://www.ncbi.nlm.nih.gov/nucleotide/751372783?report=genbank&log$=nucltop&blast_rank=8&RID=EX5FUK53013) |
| Enterobacter aerogenes strain HS112625 plasmid pHS112625 TraI (traI) gene, complete cds | 3317 | 3317 | 4% | 0 | 91% | [KJ210593.1](https://www.ncbi.nlm.nih.gov/nucleotide/608617428?report=genbank&log$=nucltop&blast_rank=9&RID=EX5FUK53013) |
| Klebsiella pneumoniae strain HS092839 plasmid pHS092839 TraI (traI) gene, complete cds | 3314 | 3314 | 4% | 0 | 91% | [KJ210594.1](https://www.ncbi.nlm.nih.gov/nucleotide/608617430?report=genbank&log$=nucltop&blast_rank=10&RID=EX5FUK53013) |
| Klebsiella pneumoniae subsp. pneumoniae strain HS092839 plasmid pHS092839, partial sequence | 1467 | 2410 | 2% | 0 | 96% | [KF724506.1](https://www.ncbi.nlm.nih.gov/nucleotide/576201388?report=genbank&log$=nucltop&blast_rank=11&RID=EX5FUK53013) |
| Escherichia coli strain M3, complete genome | 1079 | 1400 | 1% | 0 | 95% | [CP010183.1](https://www.ncbi.nlm.nih.gov/nucleotide/1114239495?report=genbank&log$=nucltop&blast_rank=12&RID=EX5FUK53013) |
