# Supplementary material for: Genome analysis of E. coli isolated from Crohn’s disease patients
Source: BMC Genomics. 2017 Jul 19;18:544. doi: 10.1186/s12864-017-3917-x (PMC5517970; doi:10.1186/s12864-017-3917-x)
Supplement: Supplementary file 9 — Test of non randomness of the operon enrichment. (DOC 871 kb) [file 12864_2017_3917_MOESM9_ESM.doc]

**Additional file 9.**

**Test of non randomness of the operon enrichment**

To verify if observed differences in OG composition between commensal strains and those isolated from patients could be attributed to chance only we performed statistical test.

First, we assume that no matter what strains are considered as commensal or CD-associated and make 1000 reshufflings of strains to group (commensal of CD) assignment and calculated Fisher test p-values for each iteration. We observe that only 25 out of 1000 reshufflings resulted in the number of OG`s with p-value < 0.05 greater or equal than 379, an observed number of OG`s with p-value < 0.05, with the mean number for all reshufflings being 108.457. So we can conclude that the probability of observing an actual number of enriched OG`s (prior to multiple comparison adjustment) is quite low (0.025) but still about 100 of OG`s are expected to be false positives. R script for performing this test is available at GitHub

[DOI: 10.5281/zenodo.546444, ogEnrichment/ reshufledStrainPvalue.r]

In order to reduce the number of false positives we compared actual gene distribution having p-value less than 0.05 with the random one. As a reference genome we used Escherichia coli LF82. Operon structure of the genome was inferred de-novo using DOOR [X. Mao, Q. Ma, C. Zhou, X. Chen, H. Zhang, J. Yang, F. Mao, W. Lai and Y Xu. DOOR 2.0: DOOR 2.0: presenting operons and their functions through dynamic and integrated views. Being reviewed by NAR Special Database Issue, 2013].

Then 10000 reshufflings between genes and operons assignment were done. For each of this reshufflings we calculated dependence of number of genes in operons with p-value < 0.05 and number of genes in operon. Visualization and table with comparison of observed data and random data obtained by reshuffling can be found at Figure 1 (below) and Additional File 9B. Operons which have observed number of genes with p-value < 0.05 greater than the maximum number of genes with p-value < 0.05 in reshuffled data were considered as highly statistically significantly overrepresented. All code needed to perform the test is available at GitHub [DOI: 10.5281/zenodo.546444, operonPval]

Figure 1 (Add_file_9A). Comparison of observed frequency of genes with p-value < 0.05 in operons with those obtained by random assignment of genes to operons. Dashed green line indicates maximum values for random data, blue dots represents operons which we consider as nonrandomly overrepresented. They are : glyoxilate metabolism - gcx part of ptn-cgl-gcx-ibe iperon, capsular assembly PAI IV LF82, iron uptake operon I, sorbose uptake and utilization, prophage I LF82, propanediol utilization operon

Additional File 9B.

| **number of genes in operon** | **Operon ID** | **observed fraction of genes with p-value < 0.05** | **mean fraction of genes with p-value < 0.05 (random)** | **max fraction of genes with p-value < 0.05 (random)** | **operon** | **OG** | **product** |
| --- | --- | --- | --- | --- | --- | --- | --- |
| 4 | 1759 | 1.00 | 0.03 | 0.75 | glyoxilate metabolism - gcx part of ptn-cgl-gcx-ibe iperon | OG0005163 | Glycerate kinase |
|  |  |  |  |  |  | OG0005162 | 2-hydroxy-3-oxopropionate reductase |
|  |  |  |  |  |  | OG0005161 | Hydroxypyruvate isomerase |
|  |  |  |  |  |  | OG0005160 | Glyoxylate carboligase |
| 6 | 1187 | 0.83 | 0.02 | 0.67 | capsular assembly PAI IV LF82 | OG0004132 | Arabinose 5-phosphate isomerase KpsF |
|  |  |  |  |  |  | OG0003925 | Vi polysaccharide export inner membrane protein VexD |
|  |  |  |  |  |  | OG0003862 | Polysialic acid transport protein KpsD precursor |
|  |  |  |  |  |  | OG0003861 | 3-deoxy-manno-octulosonate cytidylyltransferase |
|  |  |  |  |  |  | OG0003924 | Capsule polysaccharide biosynthesis protein |
|  |  |  |  |  |  | OG0003813 | Capsule polysaccharide biosynthesis protein |
| 6 | 1390 | 1.00 | 0.02 | 0.67 | iron uptake operon I | OG0003850 | Hemin-binding periplasmic protein HmuT precursor |
|  |  |  |  |  |  | OG0003917 | Oxygen-independent coproporphyrinogen-III oxidase 1 |
|  |  |  |  |  |  | OG0003918 | putative heme utilization carrier protein HutX |
|  |  |  |  |  |  | OG0003919 | Putative NADH-flavin reductase |
|  |  |  |  |  |  | OG0003881 | Hemin transport system permease protein HmuU |
|  |  |  |  |  |  | OG0003027 | Hemin import ATP-binding protein HmuV |
| 7 | 1619 | 0.86 | 0.03 | 0.57 | sorbose uptake and utilization | OG0002880 | Sorbitol dehydrogenase |
|  |  |  |  |  |  | OG0003914 | PTS system mannose-specific EIID component |
|  |  |  |  |  |  | OG0003913 | PTS system mannose-specific EIIC component |
|  |  |  |  |  |  | OG0003912 | Sorbose-specific phosphotransferase enzyme IIB component |
|  |  |  |  |  |  | OG0003911 | EIIAB-Man |
|  |  |  |  |  |  | OG0003910 | Levodione reductase |
|  |  |  |  |  |  | OG0003909 | Sor operon activator |
| 16 | 379 | 0.56 | 0.02 | 0.31 | prophage I LF82 | OG0010646 | hypothetical protein |
|  |  |  |  |  |  | OG0004889 | hypothetical protein |
|  |  |  |  |  |  | OG0007301 | hypothetical protein |
|  |  |  |  |  |  | OG0005173 | hypothetical protein |
|  |  |  |  |  |  | OG0004733 | hypothetical protein |
|  |  |  |  |  |  | OG0007276 | P22 coat protein - gene protein 5 |
|  |  |  |  |  |  | OG0007275 | hypothetical protein |
|  |  |  |  |  |  | OG0004890 | P22 tail accessory factor |
|  |  |  |  |  |  | OG0004799 | Phage stabilisation protein |
|  |  |  |  |  |  | OG0004720 | chromosome segregation protein SMC |
|  |  |  |  |  |  | OG0005169 | head assembly protein |
|  |  |  |  |  |  | OG0004586 | hypothetical protein |
|  |  |  |  |  |  | OG0004399 | hypothetical protein |
|  |  |  |  |  |  | OG0004525 | hypothetical protein |
| 19 | 833 | 0.74 | 0.03 | 0.21 | propanediol utilization operon | OG0004344 | Propanediol utilization protein PduB |
|  |  |  |  |  |  | OG0004607 | Propanediol dehydratase large subunit |
|  |  |  |  |  |  | OG0004678 | Propanediol dehydratase medium subunit |
|  |  |  |  |  |  | OG0004679 | Propanediol dehydratase small subunit |
|  |  |  |  |  |  | OG0004680 | Diol dehydratase-reactivating factor large subunit |
|  |  |  |  |  |  | OG0004681 | hypothetical protein |
|  |  |  |  |  |  | OG0004757 | hypothetical protein |
|  |  |  |  |  |  | OG0004133 | Phosphate propanoyltransferase |
|  |  |  |  |  |  | OG0004758 | putative propanediol utilization protein PduM |
|  |  |  |  |  |  | OG0004408 | Carbon dioxide concentrating mechanism protein CcmL |
|  |  |  |  |  |  | OG0004295 | Aldehyde-alcohol dehydrogenase |
|  |  |  |  |  |  | OG0003993 | Aldehyde-alcohol dehydrogenase |
|  |  |  |  |  |  | OG0004682 | Nitrogen fixation protein RnfC |
|  |  |  |  |  |  | OG0004759 | hypothetical protein |
|  |  |  |  |  |  | OG0004760 | Propanediol utilization protein PduU |
|  |  |  |  |  |  | OG0004608 | Propanediol utilization protein PduV |
