# Supplementary material for: Genome analysis of E. coli isolated from Crohn’s disease patients
Source: BMC Genomics. 2017 Jul 19;18:544. doi: 10.1186/s12864-017-3917-x (PMC5517970; doi:10.1186/s12864-017-3917-x)
Supplement: Supplementary file 11 — Bacteriocin production test in CD-associated strains. (DOC 42 kb) [file 12864_2017_3917_MOESM11_ESM.doc]

**Additional file 11**. Bacteriocin production test in CD-associated strains.

| **Sample** | **Indicator strain** | **Induction of bacteriocin production** | **Diameter of indicator inhibition zone, mm (average of 5 repeats)** | **Inhibition intensity** | **Number of Bacteriocin/Toxin OGs in genome** |
| --- | --- | --- | --- | --- | --- |
| RCE10 | MG1655 |  | 11.2 | Weak | 20 |
|  |  | UV | 12.2 | Weak |  |
|  | K12 |  | 19.2 | Strong |  |
|  |  | UV | 20.2 | Strong |  |
| RCE11 | MG1655 |  | 11.2 | Weak | 29 |
|  |  | UV | 12.2 | Weak |  |
|  | K12 |  | 17.6 | Strong |  |
|  |  | UV | 22.2 | Strong |  |
| RCE06-3 | MG1655 |  | 12.2 | Weak | 34 |
|  |  | UV | 7.8 | Weak |  |
|  | K12 |  | 10.6 | Strong |  |
|  |  | UV | 9.2 | Weak |  |
| RCE04-2 | MG1655 |  | 0 |  | 23 |
|  |  | UV | 0 |  |  |
|  | K12 |  | 8 | Weak |  |
|  |  | UV | 0 |  |  |
