# Supplementary material for: Genome analysis of E. coli isolated from Crohn’s disease patients
Source: BMC Genomics. 2017 Jul 19;18:544. doi: 10.1186/s12864-017-3917-x (PMC5517970; doi:10.1186/s12864-017-3917-x)
Supplement: Supplementary file 12 — Competition co-culture of CD-E. coli and isolates from healthy individuals. (DOC 56 kb) [file 12864_2017_3917_MOESM12_ESM.doc]

**Additional file 12.** Competition co-culture of CD-*E. coli* and isolates from healthy individuals.

CD-isolate RCE06-01 was co-cultured with two clinical isolates from healthy individuals – one from ileum of patient with no observed endoscopic pathology (healthy-2), other from the stool of a healthy volunteer (healthy-2). Liquid cultures in LB medium of equal OD 600nm (0.4) mere mixed 1:1 and mixes were inoculated into fresh LB at dilution of 1:1000.

7 re-inoculations of these cultures were made with 24h intervals at the same dilutions (inoculation 1 is the initial mixture of competing strains).

DNA from achieved cultures was extracted by CTAB method (http://www.bio-protocol.org/e97) with slight modifications.

Content of RCE06-1 DNA was evaluated by RT-PCR.

Primers used for evaluation of total E. coli DNA in sample (from Chern et al., 2010)

uidA405F CAACGAACTGAACTGGCAGA

uidA405R CATTACGCTGCGATGGAT

Custom primers used for quantity evaluation of RCE06-1 DNA in sample

(product on RCE06-01 DNA 194 bp, no product on the healthy-1 and -2 matrix)

yihU-F CCACGCATGCAATTCTGGTC

yihV-F CCGAGAAATGCAATGGTTGC

Fig. 1 presents delta Ct = Ct (RCE06 primers) – Ct (*E. coli* primers) on vertical axis, versus 8 re-inoculations (horizontal axis). All measurements are averaged values of two biological repeats.


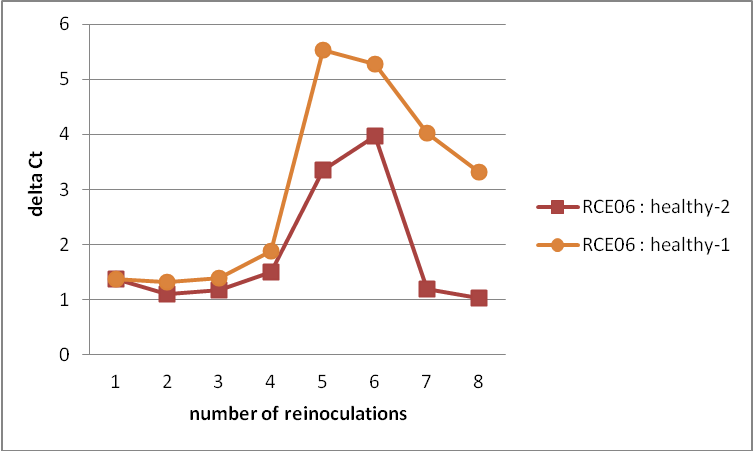


Fig. 1. RT-PCR analysis of changes in content of CD-*E. coli* DNA during co-coltivation with isolates from healthy individuals.

In both co-inoculation experiments for initial 1-3 passages content of RCE06-1 DNA is not far from initial, then it drops for some passages (reflected by increased deltaCt), then is restored again, in one case to the initial level.

Despite the data variations depending competing strain, the conclusion is definite – in standard co-cultivation conditions (LB media, 37 C, 180 RPM rotation) isolate from CD patient was unable to outcompete isolates from healthy individuals.

1. Chern EC, Siefring S, Paar J, Doolittle M, Haugland RA. Comparison of quantitative PCR assays for Escherichia coli targeting ribosomal RNA and single copy genes. Lett Appl Microbiol. 2011;52(3):298-306. doi: 10.1111/j.1472-765X.2010.03001.x.
